# MADRID+90 study on factors associated with longevity: Study design and preliminary data

**DOI:** 10.1371/journal.pone.0251796

**Published:** 2021-05-17

**Authors:** Miguel A. Fernández-Blázquez, Teodoro del Ser, Belén Frades-Payo, Marina Ávila-Villanueva, Meritxell Valentí-Soler, María T. Benítez-Robredo, Antonio Bermejo-Aguña, Eduardo J. Pedrero-Pérez, Javier Quilis-Sancho, Ana B. Pastor, Concepción Fernández-Garrido, Sara Morales-Alonso, José M. Diaz-Olalla, Nadine Correia Santos, Fernando Maestú, Jaime Gómez-Ramírez

**Affiliations:** 1 Alzheimer Disease Research Unit, CIEN Foundation, Carlos III Institute of Health, Queen Sofía Foundation Alzheimer Center, Madrid, Spain; 2 Department of Experimental Psychology, Complutense University of Madrid (UCM), Pozuelo de Alarcón, Madrid, Spain; 3 Evaluation and Quality Department, Madrid Salud, Madrid City Council, Madrid, Spain; 4 Municipal Statistics Service, S.D.G. for Statistics, Madrid City Council, Madrid, Spain; 5 Life and Health Sciences Research Institute (ICVS), School of Medicine, University of Minho, Braga, Portugal; 6 Laboratory of Cognitive and Computational Neuroscience (UCM-UPM), Center for Biomedical Technology, Pozuelo de Alarcón, Madrid, Spain; UCIBIO-REQUIMTE, Faculty of Pharmacy, University of Porto, PORTUGAL

## Abstract

The progressive aging of the population represents a challenge for society. In particular, a strong increase in the number of people over 90 is expected in the next two decades. As this phenomenon will lead to an increase in illness and age-related dependency, the study of long-lived people represents an opportunity to explore which lifestyle factors are associated with healthy aging and which with the emergence of age-related diseases, especially Alzheimer’s type dementia. The project “Factors associated with healthy and pathologically aging in a sample of elderly people over 90 in the city of Madrid” (MADRID+90) brings together a multidisciplinary research team in neurodegenerative diseases that includes experts in epidemiology, neurology, neuropsychology, neuroimaging and computational neuroscience. In the first phase of the project, a stratified random sampling was carried out according to the census of the city of Madrid followed by a survey conducted on 191 people aged 90 and over. This survey gathered information on demographics, clinical data, lifestyles and cognitive status. Here, the main results of that survey are showed. The second phase of the project aims to characterize individual trajectories in the course of either healthy and pathological aging, from a group of 50 subjects over 90 who will undergo a comprehensive clinical examination comprised of neurological and cognitive testing, MRI and EEG. The ultimate goal of the project is to characterize the biophysical and clinical profiles of a population that tends to receive little attention in the literature. A better understanding of the rapidly increasing group of nonagenarians will also help to design new policies that minimize the impact and future social and economic consequences of rapidly aging societies.

## Introduction

The progress made during the 20th century at the social, environmental and health care levels has led to a general improvement in the health status of the population and, consequently, to a reduction in mortality and a marked increase of life expectancy, which is expected to keep growing in the coming decades. In fact, the oldest-old subjects (i.e. individuals aged 90+) will number more than 70 million worldwide in 2050, a 5-fold increase over the current number of this population [1, 2]. However, this increment may also lead to an unprecedented increase in the number of people suffering from age-related diseases. In such a scenario, dementia is predicted to occupy an important and unwelcoming place. The economic, social and personal costs related to dementia are devastating not only for patients but also for their relatives. The prevalence of dementia after age 90 is projected to increase to 40% of the total oldest-old population [3].

However, still a considerable percentage of individuals remain cognitively normal at very advanced age. This fact underlies the importance of identifying protective factors for cognitive impairment in older age to set up and expand prevention strategies. For example, evidence suggests that longevity is associated with a heterogeneous set of factors such as genetic background [4], physical exercise, especially when the Body Mass Index is between 20 and 25 kg/m2 [5], effectiveness in managing daily stress [6], social support [7], having a purpose in life [8], first-degree relatives of advanced age [9], *ApoE* ε2 genotype [10], cardiovascular risk factors [11], diet quality [12], and others not yet well established. Thus, it should be noted that the main non-genetic risk factors for the development of dementia are related to potentially modifiable habits and lifestyles.

Understanding and controlling the factors that promote or reduce longevity is probably the best way to manage the economic and social consequences associated with the steady increase in life expectancy. However, the identification of the complex and interdependent mechanisms related to life extension will require the construction of a framework integrating disciplines as disparate as genetics, medicine, psychology, sociology, epidemiology, demography and economics. Only a multidisciplinary approach can provide an adequate appraisal of such a complex phenomenon as healthy aging and longevity.

On the other hand, predictive medicine aims to find out the potential diseases that an individual could develop in the future in order to conceive early preventive interventions and treatments that could prevent those same diseases before their onset. The integration of data mining and computing techniques in the form of algorithms fed with clinical, cognitive, genetic, demographic, and lifestyle data can be able to identify a series of markers to estimate the risk of an individual to suffer a particular disease and to guide individualized preventive programs.

The project “Factors associated with healthy and pathologically aging in a sample of elderly people over 90 in the city of Madrid” (MADRID+90) has a genuine interdisciplinary approach with the mission of fostering the exchange of ideas between researchers working in the fields of neurosciences, epidemiology, healthy aging and clinical pathology. MADRID+90 does not start from scratch but it is rather a continuation of previous efforts studying cognitive aging through The Vallecas Project, a longitudinal investigation for the early detection of Alzheimer’s disease (AD) in which an annual follow-up is carried out on all participants, including neurological examination, neuropsychological evaluation, brain magnetic resonance imaging (MRI) and blood analysis [13, 14]. The combination of the data obtained in both projects -MADRID+90 and The Vallecas Project- will become a very valuable tool for the identification of factors of healthy and pathological aging.

### Specific aims of MADRID+90

The primary objectives of MADRID+90 are the following:

1. To explore the phenotype of the cognitively normal oldest-old.
2. To examine factors associated with successful aging and longevity.
3. To validate a meaningful set of clinical, cognitive and functional assessment instruments for the oldest-old.
4. To determine the prevalence rates of dementia in people over 90 years according to their demographic and biological characteristics.
5. To identify modifiable risk factors that may reduce the prevalence of dementia and increase the quality of life of older people.

The present paper describes the methodology of MADRID+90 and reports the results of the initial Wave 1 assessment to better understand the living conditions of this segment of the population.

## Material and methods

### Study design

MADRID+90 is a multicentre, observational, two-Wave, cross-sectional study that will be conducted in two different Waves as described below:

- Wave 1—survey of persons aged 90 and over in the city of Madrid, Spain. The sample was randomly selected from the municipal census of Madrid which includes any person officially residing in the city. A reliable informant was always invited to be present during the conduct of the interview. Only in case the participants could not or did not know how to answer an item, the collaboration of the informants was requested and their responses were coded in the database. This Wave finished by the end of February 2020.
- Wave 2—face-to-face evaluations which were expected to end by September 2020, but due to the COVID-19 pandemic are being delayed. A subsample of 50 participants from Wave 1, with apparently preserved cognition, will be selected to complete a comprehensive clinical examination (physical and neurological exam, cognitive assessment, MRI, and EEG). The estimated time to complete the entire examination is 3 to 4 hours with convenient breaks. A reliable informant will also be interviewed to complement and verify clinical and demographic information.

### Participants

The sample was randomly selected from the municipal census of the city of Madrid, Spain, at the end of September 2019. Only two inclusion criteria were considered: (i) age 90 years or older; and (ii) individuals should be independent and home-dwelling, therefore, non-institutionalized. Recruitment and assessment of Wave 1 participants took place between October 2019 and February 2020. Registration in the municipal census is compulsory in Spain, and this is a database managed by the city councils. On January 1, 2019, 47,303 people aged 90 and over were registered in the city of Madrid, which represents 1.5% of the city’s total population. Based on a 95% confidence level and a 7% margin of error, a sample size of 200 participants was considered sufficient.

**Fig 1:**
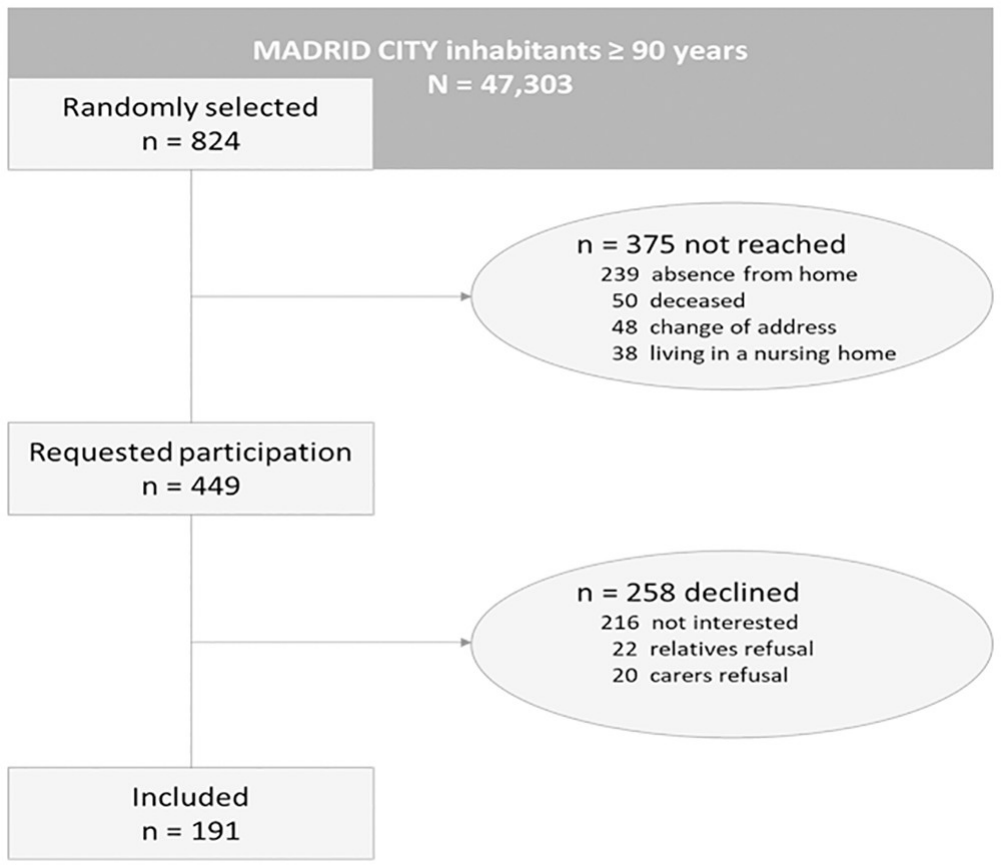
MADRID+90 flowchart.

A total of 824 non-institutionalised individuals were initially and randomly selected from the census and sent a letter inviting them to participate in the study. Then, two weeks later, interviewers were sent to the homes of the candidates to ascertain their willingness to participate in the study. Of the 824 individuals invited to participate, 375 were not reached due to absence from home, change of residence or death. From the remaining 449 who were contacted to confirm eligibility, 258 declined after further information on the study. The final sample comprised 191 individuals who were interviewed at their homes with the presence, when possible, of a reliable informant. Fig 1 shows the flowchart of MADRID+90. The study was approved by the Ethics Committee of the Carlos III Institute of Health. Prior to inclusion, all individuals gave oral informed consent to participate in Wave 1.

### Assessments

The Wave 1 surveys were conducted at the participants’ homes by trained research staff. The complete visit took approximately 15 to 30 minutes. A structured questionnaire mainly based on previous population surveys of the Madrid City Council was used to collect information related to the following areas:

- Demographic data: Age, gender, education, prior occupation, marital status, life setting, social network, birth place, home characteristics.
- Morbidity: A comprehensive list of highly prevalent age-associated diseases is presented for the subject to confirm whether he/she has a formal diagnosis in some of them by a physician (coded as Yes/No).
- Medication: The participant is asked to list all the drugs he/she takes on a daily basis.
- Subjective cognitive decline (SCD): This construct refers to a self-experienced persistent decline in cognitive abilities in comparison with a previously normal status and independently of the objective performance on neuropsychological tests. Our study uses a filter question that is simple to understand and answer for the oldest-old ("Do you have memory problems?"). If the participant responds "yes" six other related questions are administered to analyze the characteristics of his or her subjective perception of cognitive decline.
- Spanish version of the Telephone Interview for Cognitive Status (TICS): This is a screening test to measure cognitive status that is usually administered by phone and offers better performance than Mini Mental State Examination, with less ceiling effect and high accuracy in detecting mild cognitive impairment and dementia [15, 16]. In our case, due to the sensory limitations that characterized the oldest-old, we decided to adapt the scale so that it could be applied face-to-face during the interview.
- Perceived health and quality of life: A self-perceived health item, a health thermometer (range 0 to 100), and the health-related quality of life measured through the 9-dimensions COOP/WONCA scale [17] were administered.
- Functional dependence: Several items were presented to assess disability and dependency status (officially certified by the public administration), sensory hearing and visual impairment, as well as the Katz index of independency in activities of daily living [18].
- Lifestyle: Weight, height, hours and quality of sleep, alcohol and tobacco consumption, dietary intake related to the Mediterranean diet, and frequency of daily and leisure activities.

In supplemental S1 and S2 Files we provide the full questionnaire used in this survey, the original in Spanish and the one translated into English, so that the reader can see in more detail the variables used in the study. The responses of the participants, and the proxies when appropriate, were directly coded in electronic devices, so that the information was uploaded directly into the database. This procedure ensures data traceability and increases the quality of the information obtained.

On the other hand, the Wave 2 protocol is being applied in face-to-face visits in a subsample of 50 individuals recruited in Wave 1 and presumably without dementia. The protocol will include the following measures:

#### Clinical and neurological exam

A semi-structured clinical interview focused on neurological diseases, psychiatric disorders and family history of dementia is being conducted by a neurologist. A medical exam is also collecting systolic and diastolic blood pressure, heartbeat, reflexes, grip strength, signs of parkinsonism as well as the performance in brief motor tasks [19, 20]. During the visit, the neurologist estimates visual, hearing and mobility functions which are classified as ‘‘good”, ‘‘moderate”, ‘‘poor” or ‘‘very poor”, according to a subjective classification previously used [21]. Additionally, for females age at menarche, onset of menopause, number of pregnancies and/or miscarriages are also collected.

#### Cognitive assessment

A comprehensive neuropsychological battery is administered by an experienced neuropsychologist to assess various cognitive domains important for the diagnosis of dementia and pre-dementia stages:

- Cognitive status: Mini Mental State Examination (MMSE) [22]; Clinical Dementia Rating (CDR) [23].
- Processing speed: Digit Symbol Coding [24].
- Verbal episodic memory: Free and Cued Selective Reminding Test (FCSRT) [25].
- Attention / executive functions: Digit span Forward and Backward [24]; Frontal Assessment Battery (FAB) [26].
- Language: Lexical-Semantic fluency (letter P and animals in one minute) [27]; repetition of sentences [28]; Boston Naming Test 15-items version [29].
- Visuospatial: Incomplete Letter from the Visual Object and Space Perception (VOSP) Battery [30].
- Visuoconstructive skills: Copy of Figures from the Revised Barcelona Test [28]; Clock Drawing Test [31].

In addition, the cognitive assessment is completed with specific scales on SCD [13, 32], depression [33], anxiety [34, 35], and functional state [36].

#### MRI scan

All participants in Wave 2 are invited to undergo a brain MRI scan. Those who agree will be screened for contraindications (pacemaker, metallic implant or foreign bodies, ferromagnetic clips, claustrophobia, etc.). All scans are carried out in a 3-T MRI (Signa HDxt GEHC, Waukesha, WI, USA) equipped with a gradient system of 50 mT/m. A phased array eight channels brain coil will be used for all the participants. The neuroimaging protocol includes the following sequences: (i) 3D T1-weigthed structural: 3D FSPGR with IR preparation (TR 10 ms, TE 4.5 ms, TI 600 ms), field of view FOV 256 mm, matrix 288 × 288, slice thickness 1 mm with no gap between; (ii) FLAIR: axial 2D FSE IR (repetition time TR 9000 ms, echo time TE 130 ms, TI 2100 ms), FOV 256 mm, slice thickness 3.4 cm with no gap between; (iii) T2-weighted: axial 2D GRE EPI (TR 3475 ms, TE 27 ms), FOV 256 mm, matrix 262 × 192, slice thickness 3 mm with no gap between; (iv) diffusion weighted imaging (DWI): single shot spin-echo echo-planar imaging sequence DWI SE-EPI (TR 9200 ms, TE 80 ms) with a-value of 800 s/mm2 and 21 gradient directions FOV 256 mm, matrix 128 × 128, slice thickness 3 mm with no gap between; (v) resting state (rs-fMRI): acquisition with blood oxygen level dependent (BOLD), without task, during 10 minutes (TR 2500 ms, TE 27 ms), FOV 250 mm, matrix 96 × 96, slice thickness 3 cm with no gap between; and (vi) GABA spectroscopy: voxel of interest VOI = 20×20×20 mm^3^ placed over prefrontal and precuneus areas using a STEAM sequence (TE/TM/TR = 17/17/2000 ms, sample size = 4096, spectral bandwidth = 4000 Hz, phase cycling = 8, 288 averages). The total time of each subject’s scanning session is approximately 50 minutes.

#### EEG recording

EEG is recorded simultaneously from 32 channels corresponding to the International 10–20 System. Left and right earlobes (A1 and A2) are marked as reference electrodes. EEG data are recorded under resting state condition. Resting EEG recordings are obtained under eyes open and eyes closed for 5 minutes each. The artefacts in the EEG signal are removed using simultaneous low-pass filtering and total variation denoising. The total time of each EEG session is approximately 40 minutes.

### Planned statistical analyses

Data shall be recorded in a standardized and relational database. When all data from Waves 1 and 2 have been collected a strategy based on data mining techniques will be used for the purpose of comparing the information collected. Different models will be developed based both on classical parametric methods (linear models, logistic regression, etc.) and on supervised and unsupervised automatic learning algorithms (vector support machines, clusters, Bayesian classifiers, deep neural networks, etc.). The planned analysis strategy will be carried out in three different blocks depending on the information to be compared and the objectives to be met:

1. **Analysis of the information obtained from the survey of Wave 1**: This analysis first aims to find out living conditions and to identify potential factors associated with successful and longevity. Subsequently, based on the scores obtained by the participants of MADRID+90 on the TICS scale, the sociodemographic and lifestyle characteristics that differentiate the subjects with the best cognitive performance from those with the worst performance will be analyzed. The analysis of all these factors will be carried out taking into account the temporal dynamics of their incidence on healthy aging, as well as the mutual interactions between all of them.
2. **Analysis of the information obtained from the face-to-face cognitive evaluations of Wave 2**: This analysis will provide evidence to adapt and validate for the Spanish population a battery of clinical, cognitive and functional examination applicable to people 90+ years of age. This constitutes an important milestone in research on aging insofar as it will provide specific assessment instruments for a segment of the population that is expected to increase substantially in the coming years.
3. **Comparative analysis of the information obtained in the complete sample of MADRID+90 with that of The Vallecas Project**: MADRID+90 will join synergies with The Vallecas Project for the early detection of AD [13] so that the conclusions obtained will be enriched. The comparison of data from both Waves of MADRID+90 together with the cohort of The Vallecas Project will allow to deepen the knowledge of the factors associated with healthy aging, as well as to better understand the markers of dementia risk. To this end, the MADRID+90 sample will act as a control group over which to compare two different subgroups of The Vallecas Project: i) individuals diagnosed as cognitively healthy in all their visits; and ii) individuals who have developed dementia throughout the project. We will specifically compare sociodemographic, clinical and lifestyle variables, as well as their temporal dynamics, which may predict successful aging and the risk of developing dementia. The ultimate goal of these analyses is to obtain an algorithm that can predict the progressive course in healthy and pathological aging.

In the present work, a simple preliminary descriptive analysis of the data collected in Wave 1 was carried out. For this purpose, robust statistics were provided (median and interquartile range) for ordinal and quantitative variables, while frequencies and percentages were obtained for categorical variables. Moreover, to the extent that gender might play an important role in the field of longevity, potential differences between males and females were analysed for all the variables considered. Thus, Chi-square and Fisher tests (for 2x2 tables) were calculated in the case of categorical variables and Wilcoxon non-parametric tests for quantitative variables. An additional analysis was carried out to compare the cognitive performance of demented and non-demented participants in the TICS test. Given the exploratory nature of all these analyses, we did not considered the need to control for different covariates or to correct for multiple comparisons. A p-value of 0.05 was used as the threshold for statistical significance. All analyses were conducted using R version 4.0.0.

## Results

The sociodemographic characteristics of the sample are shown in Table 1. The median age of the sample was 92.64 years (range: 90–102), 72.8% females, and 79.1% widowed. The predominant educational attainment achieved by the sample was primary education (52.4%). When asked about occupation 48.2% of the participants had a prior occupational status equivalent to unskilled manual labour. Regarding living conditions, 23.6% lived alone, 52.9% with spouse and 23.6% with other accompanied. Statistically significant differences were found between males and females, with males being more educated and having more skilled work, while females were older as well as widowed in a greater proportion than males (p<0.001).

**Table 1:** Sociodemographic characteristics of the sample.

	TOTAL (N = 191)	FEMALE (n = 139)	MALE (n = 52)	p-value
**Age (median [IQR**^a^**])**	92.64 [91.61, 94.89]	93.01 [91.74, 95.32]	91.88 [91.45, 94.44]	0.027
**Sex (%)**				
** Female**	139 (72.8)			
** Male**	52 (27.2)			
**Education (%)**				
** Less than Primary education**	32 (16.8)	28 (20.1)	4 (7.7)	0.021
** Primary education**	100 (52.4)	74 (53.2)	26 (50.0)	
** Secondary education**	49 (25.7)	33 (23.7)	16 (30.8)	
** Higher education**	10 (5.2)	4 (2.9)	6 (11.5)	
**Prior occupation (%)**				
** Management**	15 (7.9)	6 (4.3)	9 (17.3)	<0.001
** Professional**	18 (9.4)	10 (7.2)	8 (15.4)	
** Office clerk**	19 (9.9)	14 (10.1)	5 (9.6)	
** Skilled manual labor**	47 (24.6)	25 (18.0)	22 (42.3)	
** Unskilled manual labor**	92 (48.2)	84 (60.4)	8 (15.4)	
**Marital status (%)**				
** Single**	10 (5.2)	9 (6.5)	1 (1.9)	<0.001
** Married**	30 (15.7)	7 (5.0)	23 (44.2)	
** Widowed**	151 (79.1)	123 (88.5)	28 (53.8)	
**Life setting (%)**				
** Alone**	45 (23.6)	35 (25.2)	10 (19.2)	0.663
** Spouse**	101 (52.9)	71 (51.1)	30 (57.7)	
** Other accompanied**	45 (23.6)	33 (23.7)	12 (23.1)	

^a^IQR: Interquartile range.

### Health status and morbidities

Based on the diagnostics collected, the sample had on average symptoms of 3 morbidities (IQR: 2–4) and 3 prescribed drugs (IQR: 2–5). Hypertension was the most common vascular risk factor, with 68.1% of the sample having been diagnosed with it. The rate of previously diagnosed hypercholesterolemia and diabetes mellitus was 13.6% and 19.4% respectively. Heart disease was found in 6.8% of the sample and past stroke was reported by 5.8%. Overall, no significant differences were found between males and females in the diagnosis of cardiovascular disease, although there was a higher tendency among males to suffer from this type of morbidity (Table 2). Musculoskeletal disease (arthrosis / osteoporosis) was however much more prevalent in females than males (81.3% vs. 38.5%; p < 0.001). 24% of the sample was diagnosed with some type of dementia. Interestingly, the rate of cancer was 5.8% for both males and females, being breast, colon, prostate and uterus the most prevalent forms of cancer.

**Table 2:** Clinical characteristics of the sample.

	TOTAL (N = 191)	FEMALE (n = 139)	MALE (n = 52)	p-value
**Hypertension (%)**				
** No**	61 (31.9)	45 (32.4)	16 (30.8)	0.864
** Yes**	130 (68.1)	94 (67.6)	36 (69.2)	
**Hypercholesterolemia (%)**				
** No**	165 (86.4)	123 (88.5)	42 (80.8)	0.234
** Yes**	26 (13.6)	16 (11.5)	10 (19.2)	
**Diabetes (%)**				
** No**	154 (80.6)	115 (82.7)	39 (75.0)	0.303
** Yes**	37 (19.4)	24 (17.3)	13 (25.0)	
**Heart disease**^a^ **(%)**				
** No**	178 (93.2)	132 (95.0)	46 (88.5)	0.192
** Yes**	13 (6.8)	7 (5.0)	6 (11.5)	
**COPD**^b^ **(%)**				
** No**	169 (88.5)	123 (88.5)	46 (88.5)	1.000
** Yes**	22 (11.5)	16 (11.5)	6 (11.5)	
**Musculoskeletal disease**				
** No**	58 (30.4)	26 (18.7)	32 (61.5)	< 0.001
** Yes**	133 (69.6)	113 (81.3)	20 (38.5)	
**Depression (%)**				
** No**	163 (85.3)	116 (83.5)	47 (90.4)	0.260
** Yes**	28 (14.7)	23 (16.5)	5 (9.6)	
**Stroke (%)**				
** No**	180 (94.2)	134 (96.4)	46 (88.5)	0.073
** Yes**	11 (5.8)	5 (3.6)	6 (11.5)	
**Dementia**				
** No**	145 (75.9)	106 (76.3)	39 (75.0)	0.851
** Yes**	46 (24.1)	33 (23.7)	13 (25.0)	
**Cancer (%)**				
** No**	180 (94.2)	131 (94.2)	49 (94.2)	1.000
** Yes**	11 (5.8)	8 (5.8)	3 (5.8)	
**Number of drugs (median [IQR**^c^**])**	3.00 [2.00, 5.00]	3.00 [2.00, 5.00]	3.00 [2.00, 6.00]	0.833

^a^Here are included chronic ischemic heart disease, heart failure, and arrhythmia.

^b^COPD: Chronic obstructive pulmonary disease.

^c^IQR: Interquartile range.

### Cognitive status

**Fig 2:**
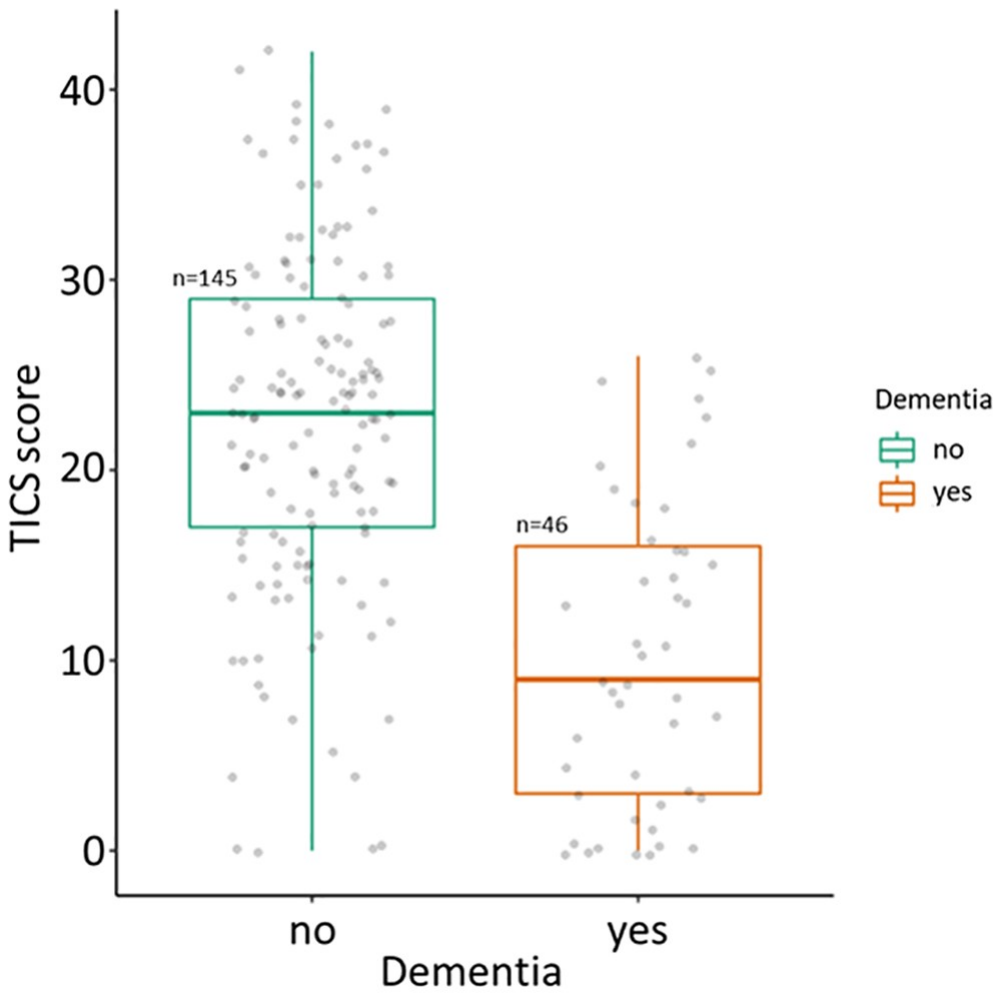
Cognitive performance in TICS by dementia diagnostic.

Approximately one third of the sample reported the presence of SCD with a median progression of 3 years (IQR: 2–5). Although 40.3% of participants showed signs of concern because of the complaints, only 34.6% reported seeking medical help for this reason. In any case, only 22.5% acknowledged that they were cognitively worse off than the rest of their age group. No significant differences were found between males and females in SCD (Table 3). Regarding cognitive performance the median of the TICS total score was 20 (IQR: 13–26.5) with males scoring only slightly higher than females (21.5 vs. 20.0; p = 0.343). Similarly, no significant differences were found between males and females in any of the TICS subtests, although a slight trend in favour of males was noted in all scores. As expected median cognitive performance in TICS for non-demented was higher and statistically significant than demented (23 vs. 9; p<0.001). Fig 2 displays the differences between both groups.

**Table 3:** Subjective and objective cognitive status of the sample.

	TOTAL (N = 191)	FEMALE (n = 139)	MALE (n = 52)	p-value
**SCD**^a^ **(%)**				
** No**	129 (67.5)	95 (68.3)	34 (65.4)	0.730
** Yes**	62 (32.5)	44 (31.7)	18 (34.6)	
**SCD years (median [IQR**^b^**])**	3.00 [2.00, 5.00]	3.00 [2.00, 5.00]	2.00 [1.00, 4.75]	0.199
**Concerns (%)**				
** No**	114 (59.7)	79 (56.8)	35 (67.3)	0.246
** Yes**	77 (40.3)	60 (43.2)	17 (32.7)	
**Medical help for SCD (%)**				
** No**	125 (65.4)	89 (64.0)	36 (69.2)	0.609
** Yes**	66 (34.6)	50 (36.0)	16 (30.8)	
**Daily impact (%)**				
** No**	120 (62.8)	87 (62.6)	33 (63.5)	1.000
** Yes**	71 (37.2)	52 (37.4)	19 (36.5)	
**Worse than others (%)**				
** No**	148 (77.5)	108 (77.7)	40 (76.9)	1.000
** Yes**	43 (22.5)	31 (22.3)	12 (23.1)	
**TICS**^c^ **Orientation (median [IQR])**	9.00 [6.00, 12.00]	9.00 [6.00, 11.00]	9.00 [5.00, 12.00]	0.868
**TICS Calculation (median [IQR])**	0.00 [0.00, 1.00]	0.00 [0.00, 1.00]	0.00 [0.00, 2.00]	0.147
**TICS Abstraction (median [IQR])**	5.00 [1.00, 8.50]	5.00 [1.00, 8.00]	5.50 [0.75, 10.00]	0.313
**TICS Immediate Memory (median [IQR])**	1.00 [0.00, 3.00]	1.00 [0.00, 3.00]	2.00 [0.00, 3.00]	0.237
**TICS Delayed Memory (median [IQR])**	0.00 [0.00, 1.00]	0.00 [0.00, 1.00]	0.00 [0.00, 2.00]	0.423
**TICS Working Memory (median [IQR])**	2.00 [0.00, 6.00]	2.00 [0.00, 4.00]	3.00 [0.00, 7.00]	0.132
**TICS Total score (median [IQR])**	20.00 [13.00, 26.50]	20.00 [13.00, 25.00]	21.50 [13.00, 32.00]	0.343

^a^SCD: Subjective Cognitive Decline.

^b^IQR: Interquartile range.

^c^TICS: Telephone Interview for Cognitive Status.

### Subjective health, quality of life and functionality

35% of the sample self-reported their general health as being good or very good versus 33.0% rating it as fair and 31.9% as bad or very bad (Table 4). The majority of the participants (91.6%) rated their quality of life as good, very good or excellent despite self-reporting 30.4% of moderate or severe usual pain and 27.2% poor to very poor sleep quality. With regard to social support, 94.2% of the sample indicated that they had help from different people if they needed it, although 36.2% of the participants frequently experienced feelings of loneliness. Rates of disability and dependency in the sample were 17.3% and 18.8% respectively, with 80.6% having moderate or good vision and 83.2% moderate or good hearing abilities. Katz Index showed that the majority of the participants remain independent for bathing (59.2%), continence (60.7%), dressing (62.3%), mobility (66.0%), toileting (67.0%), and feeding (75.9%). No significant differences by sex were found in all these variables.

**Table 4:** Self-perceived health, quality of life and functionality of the sample.

	TOTAL (N = 191)	FEMALE (n = 139)	MALE (n = 52)	p-value
**Self-perceived health (%)**				
** 1-Very good**	10 (5.2)	9 (6.5)	1 (1.9)	0.480
** 2-Good**	57 (29.8)	39 (28.1)	18 (34.6)	
** 3-Neither good nor bad**	63 (33.0)	47 (33.8)	16 (30.8)	
** 4-Bad**	22 (11.5)	18 (12.9)	4 (7.7)	
** 5-Very bad**	39 (20.4)	26 (18.7)	13 (25.0)	
**Pain (%)**				
** 1-Not at all**	42 (22.0)	25 (18.0)	17 (32.7)	0.273
** 2-Very slight**	42 (22.0)	32 (23.0)	10 (19.2)	
** 3-Mild**	49 (25.7)	36 (25.9)	13 (25.0)	
** 4-Moderate**	33 (17.3)	27 (19.4)	6 (11.5)	
** 5-Severe**	25 (13.1)	19 (13.7)	6 (11.5)	
**Social support (%)**				
** 0-None**	1 (0.5)	0 (0.0)	1 (1.9)	0.258
** 1-Someone**	10 (5.2)	8 (5.8)	2 (3.8)	
** 2-Some people**	31 (16.2)	23 (16.5)	8 (15.4)	
** 3-Many people**	63 (33.0)	50 (36.0)	13 (25.0)	
** 4-Everybody**	86 (45.0)	58 (41.7)	28 (53.8)	
**Quality of life (%)**				
** 1-Excellent**	31 (16.2)	23 (16.5)	8 (15.4)	0.885
** 2-Very good**	60 (31.4)	41 (29.5)	19 (36.5)	
** 3-Good**	84 (44.0)	63 (45.3)	21 (40.4)	
** 4-Fair**	15 (7.9)	11 (7.9)	4 (7.7)	
** 5-Poor**	1 (0.5)	1 (0.7)	0 (0.0)	
**Loneliness (%)**				
** 1-Always**	28 (14.7)	21 (15.1)	7 (13.5)	0.562
** 2-Often**	41 (21.5)	33 (23.7)	8 (15.4)	
** 3-Sometimes**	40 (20.9)	29 (20.9)	11 (21.2)	
** 4-Rarely to never**	82 (42.9)	56 (40.3)	26 (50.0)	
**Disability (%)**				
** No**	158 (82.7)	114 (82.0)	44 (84.6)	0.830
** Yes**	33 (17.3)	25 (18.0)	8 (15.4)	
**Dependency (%)**				
** No**	155 (81.2)	115 (82.7)	40 (76.9)	0.407
** Yes**	36 (18.8)	24 (17.3)	12 (23.1)	
**Vision (%)**				
** Good**	108 (56.5)	78 (56.1)	30 (57.7)	1.000
** Moderate**	46 (24.1)	34 (24.5)	12 (23.1)	
** Poor**	3 (1.6)	2 (1.4)	1 (1.9)	
** Very poor**	34 (17.8)	25 (18.0)	9 (17.3)	
**Hearing (%)**				
** Good**	108 (56.5)	77 (55.4)	31 (59.6)	0.775
** Moderate**	51 (26.7)	36 (25.9)	15 (28.8)	
** Poor**	12 (6.3)	10 (7.2)	2 (3.8)	
** Very poor**	20 (10.5)	16 (11.5)	4 (7.7)	
**KATZ Index Bath (%)**				
** Dependent**	78 (40.8)	56 (40.3)	22 (42.3)	0.869
** Independent**	113 (59.2)	83 (59.7)	30 (57.7)	
**KATZ Index Dress (%)**				
** Dependent**	72 (37.7)	54 (38.8)	18 (34.6)	0.619
** Independent**	119 (62.3)	85 (61.2)	34 (65.4)	
**KATZ Index WC (%)**				
** Dependent**	63 (33.0)	46 (33.1)	17 (32.7)	1.000
** Independent**	128 (67.0)	93 (66.9)	35 (67.3)	
**KATZ Index Mobility (%)**				
** Dependent**	65 (34.0)	49 (35.3)	16 (30.8)	0.610
** Independent**	126 (66.0)	90 (64.7)	36 (69.2)	
**KATZ Index Continence (%)**				
** Dependent**	75 (39.3)	57 (41.0)	18 (34.6)	0.506
** Independent**	116 (60.7)	82 (59.0)	34 (65.4)	
**KATZ Index Feeding (%)**				
** Dependent**	46 (24.1)	35 (25.2)	11 (21.2)	0.704
** Independent**	145 (75.9)	104 (74.8)	41 (78.8)	
**Sleep quality (%)**				
** Good to moderate**	94 (49.2)	69 (49.6)	25 (48.1)	0.532
** Intermediate**	45 (23.6)	30 (21.6)	15 (28.8)	
** Poor to very poor**	52 (27.2)	40 (28.8)	12 (23.1)	

### Lifestyle characteristics

While 12.0% of the participants had smoked at some stage in their lives (males significantly more often than females; 30.8% vs. 5.0%, p<0.001), only 1.6% currently smoke. Also, alcohol consumption was low in our sample (4.7%), with a higher trend among males (9.6%). Interestingly, no cases of past alcohol abuse were found in our sample. Regarding diet the daily consumption of fruits, vegetables and extra virgin olive oil stands out, followed by legumes and fish. No significant differences were found in any food group according to sex; only fish consumption was slightly higher among men than among women. On the other hand, walking, watching tv/listening to radio and reading were the most frequent leisure activities among the participants. According to the IQR scores (Table 5), females self-reported a higher level of sedentariness in all the leisure activities. In addition, significant differences were found in favour of men in the activities of meeting friends (p = 0.028) and use of Information and Communications Technology (ICT) (p = 0.003).

**Table 5:** Lifestyle characteristics of the sample.

	TOTAL (N = 191)	FEMALE (n = 139)	MALE (n = 52)	p-value
**Smoking (%)**				
** Currently**	3 (1.6)	2 (1.4)	1 (1.9)	<0.001
** In the past**	23 (12.0)	7 (5.0)	16 (30.8)	
** Never**	165 (86.4)	130 (93.5)	35 (67.3)	
**Alcohol (%)**				
** Currently**	9 (4.7)	4 (2.9)	5 (9.6)	0.064
** Never**	182 (95.3)	135 (97.1)	47 (90.4)	
**Vegetables (median [IQR**^a^**])**	4.00 [3.00, 4.00]	4.00 [3.00, 4.00]	4.00 [3.00, 4.00]	0.807
**Fruit (median [IQR])**	4.00 [4.00, 4.00]	4.00 [4.00, 4.00]	4.00 [4.00, 4.00]	0.686
**Extra virgin olive oil (median [IQR])**	4.00 [3.00, 4.00]	4.00 [3.00, 4.00]	4.00 [3.00, 4.00]	0.865
**Legumes (median [IQR])**	2.00 [2.00, 3.00]	2.00 [2.00, 3.00]	2.00 [2.00, 3.00]	0.170
**Fish (median [IQR])**	2.00 [1.00, 2.00]	2.00 [1.00, 2.00]	2.00 [2.00, 3.00]	0.097
**Nuts (median [IQR])**	0.00 [0.00, 2.00]	0.00 [0.00, 1.00]	0.00 [0.00, 2.00]	0.385
**Coffee (median [IQR])**	1.00 [0.00, 4.00]	1.00 [0.00, 4.00]	2.00 [0.00, 4.00]	0.392
**Walking (median [IQR])**	3.00 [0.00, 4.00]	2.00 [0.00, 4.00]	3.50 [0.00, 4.00]	0.470
**Other physical activity (median [IQR])**	0.00 [0.00, 0.00]	0.00 [0.00, 0.00]	0.00 [0.00, 0.00]	0.062
**Artistic (median [IQR])**	0.00 [0.00, 0.00]	0.00 [0.00, 0.00]	0.00 [0.00, 0.00]	0.643
**Meet friends (median [IQR])**	0.00 [0.00, 0.00]	0.00 [0.00, 0.00]	0.00 [0.00, 2.00]	0.028
**Pastime (median [IQR])**	0.00 [0.00, 0.00]	0.00 [0.00, 0.00]	0.00 [0.00, 2.00]	0.192
**Courses (median [IQR])**	0.00 [0.00, 0.00]	0.00 [0.00, 0.00]	0.00 [0.00, 0.00]	0.196
**Cinema (median [IQR])**	0.00 [0.00, 0.00]	0.00 [0.00, 0.00]	0.00 [0.00, 0.00]	0.899
**Music (median [IQR])**	0.00 [0.00, 1.00]	0.00 [0.00, 0.00]	0.00 [0.00, 1.25]	0.261
**TV / Radio (median [IQR])**	4.00 [1.00, 4.00]	4.00 [1.00, 4.00]	4.00 [3.25, 4.00]	0.681
**Reading (median [IQR])**	1.00 [0.00, 4.00]	0.00 [0.00, 4.00]	1.00 [0.00, 4.00]	0.272
**ICT**^b^ **(median [IQR])**	0.00 [0.00, 1.00]	0.00 [0.00, 0.00]	0.00 [0.00, 3.25]	0.003

^a^IQR: Interquartile range.

^b^ICT: Information and communications technology.

## Discussion

MADRID+90 represents one of the few research projects in the world specifically addressed to the oldest-old community-dwelling population. The research programme is divided into two different phases and the exploratory outcomes obtained in Wave 1 have been presented here. Thus, the main aim of this study was to describe the methodological design of MADRID+90 and to provide health, functional and living conditions profiles in 191 individuals aged 90+ randomly selected from the municipal census of the city of Madrid.

Our results showed that regarding to the sociodemographic profile, females outnumber males by representing 72.8% of the sample. Females also were older than males what is an expected result since females live longer than males with a difference in life expectancy at birth around four to six years [37]. The median number of co-morbid diseases found in our sample was 3, similar to that reported in other population-based studies [38]. Hypertension was the most common vascular risk factor, with 68.1% of the sample having been diagnosed with it. Hypertension is considered as the most common chronic disease of older adults [39] as well as a major contributor to atherosclerosis, which causes inflammation and increases risk for vascular events [40]. It was also observed higher tendency among males to suffer from cardiovascular diseases than females. On the other hand, rate of cancer for our sample was 5.8%, a very similar rate to that found in a recent population‐based cancer incidence study [41]. After cardiovascular disease, cancer is the second cause of death in older adults. Nevertheless, the risk of receiving a diagnosis of cancer varies throughout the lifespan so that from age 85 the likelihood of the diagnosis begins to decrease, as does the death rate associated with cancer [42]. Thus, after age 90 cancer is an uncommon cause of disease or death [43].

Disability rates are usually high among the oldest-old population. Previous studies have reported that the prevalence of disability increases exponentially in older adults, especially at the age of 85 [44]. In our study we found that around 60% of the sample is dependent for at least one daily living activity as proposed in the Katz Index. This finding is consistent with previous studies that have reported 21.5–66% nonagenarians with difficulties in one or more daily living activities [44, 45]. We also found that among self-care activities bathing has the highest prevalence of disability among nonagenarians, as previously reported [46, 47]. Regarding vision, visual acuity tends to decrease with age producing different types of impairments. In our sample 17% of individuals showed signs of very poor vision, a slightly better figure than that provided in other studies [48]. On the other hand, hearing loss also increases with aging. Our results showed a 10.5% of prevalence of very poor hearing that is in line with other studies that estimate a 6–7% of severe hearing loss [49, 50]. Both visual impairment and, especially, hearing loss are two of the main disabilities associated with an increased risk of social isolation, depression, dementia, and dependence. Hearing aids are underused in this population despite their potential benefits. To improve use, it has been suggested that hearing aids should be considered as a type of lifestyle modification [51].

The results of Wave 1 of MADRID+90 showed that 24.1% of individuals had been diagnosed as demented by a physician. However, distinguishing between normal and pathological aging in the oldest-old is particularly difficult. The lack of cognitive assessment tools adapted and validated for this population group, together with the lack of operational definitions of cognitive impairment at advanced ages, hinder the diagnosis in people over 90 years of age [52]. Compared to younger older adults, individuals over 90 have greater variability in their physical and mental status which increases the difficulty in interpreting their clinical, social and cognitive assessments. In addition, as a collateral effect to their exceptional longevity, there is a high percentage of people 90+ with sensory (visual or auditory) and psychomotor disabilities, which is an impediment to perform any standard neurocognitive examination [53]. Consequently, there is a clear lack of consensus on the criteria for diagnosing dementia over the age of 90 [54]. This lack of consensus can lead to notable discrepancies in epidemiological data. For example, some studies have suggested a decrease in the prevalence of dementia in very old individuals with respect to younger ages [55], obtaining dementia rates in people older than 90 years of 22.1% for men and 30.8% for women [56], which stagnate around 40% at age 95 [57], or even show a deceleration in prevalence-incidence by age [58]. On the other hand, other studies indicate that both the incidence and prevalence of dementia continue to increase in nonagenarians and centenarians [3], and may even do so at the same rate as in the less advanced age groups [59]. Specifically, in centenarians, dementia prevalence rates are even more contradictory, with percentages ranging from 25 to 100% depending on the studies [60]. All these discrepancies could be explained, at least in part, due to the lack of systematic research in the oldest-old and the extreme complexity of precisely defining the diagnostic criteria.

Effective strategies are needed to maintain the best possible health and well-being later in life. The key role played by lifestyle in the paradigm shift of longevity and aging cannot be overlooked. Variables such as diet, physical activity, economic status, education, social relationships, stress or socio-cultural context have been repeatedly investigated and are pieces of a puzzle that could help increase life expectancy and quality of life. Our results show that, although 12% of the participants had smoked at some stage in their lives, especially males, only 1.6% currently smoke. Moreover, significant alcohol consumption was very low in our sample (4.7%) with a higher tendency again among males. In terms of diet, the daily consumption of fruits, vegetables and extra virgin olive oil stands out, followed by legumes and fish. Those foods are good indicators of compliance with a Mediterranean diet, which has been repeatedly associated with longevity [61]. On the other hand, walking, watching tv/listening to radio, and reading were the most frequent leisure activities, with females self-reporting a higher level of sedentary lifestyle across all leisure activities, which could perhaps be related with their older age. Specifically, the benefits inherent in maintaining a healthy lifestyle can be classified into groups according to their susceptibility to be easily modifiable or not. This classification will be decisive in establishing individualized longevity strategies: (i) variables related to sociodemographic factors (not easily modifiable): income level, educational attainment, place of residence, marital status, employment, environmental pollution; and (ii) lifestyle-related variables (apparently more easily modifiable): nutrition, overweight, physical activity, intellectual activity, hours and quality of sleep, social network, life satisfaction, stress, and leisure activities.

## Conclusion

While the expected strong demographic change poses major challenges to societies in economic, health and ethical terms, this unprecedented situation presents itself as an opportunity to understand the determinants of healthy aging and longevity. Nonagenarians and centenarians can no longer be considered as a statistical anomaly, but as a growing segment of the population of invaluable importance for identifying key factors related to successful aging. In the next 20 years, societies will be forced to face this challenge in order to minimise its social, health and economic consequences. The study of the biopsychosocial and clinical profile of the oldest-old will help to make public health policy decisions. In addition, the study of very old individuals who have successfully aged could also help to decipher the combination of lifestyle factors, therefore modifiable, that make it possible to prevent age-related diseases, especially dementia and, mainly, AD. Ultimately, healthy aging must allow to sustain life in the best possible conditions, a long, healthy and active life, without the suffering caused by diseases that limit movement and diminish the capacity to remember the past and imagine future. A life worth living at 90, 100 and beyond, this is the mission and vision of MADRID+90.

## Supporting information

S1 File Wave 1 questionnaire in Spanish. (DOCX)

S2 File Wave 1 questionnaire in English. (DOCX)
