# Supplementary material for: MADRID+90 study on factors associated with longevity: Study design and preliminary data
Source: PLoS One. 2021 May 17;16(5):e0251796. doi: 10.1371/journal.pone.0251796 (PMC8128242; doi:10.1371/journal.pone.0251796)
Supplement: S1 File — (DOCX) [file pone.0251796.s001.docx]

**CUESTIONARIO DE LA ENCUESTA “PROGRAMA DE INVESTIGACIÓN**

**EN LONGEVIDAD ESPAÑA-PORTUGAL +90” (PILEP+90)**

**EJEMPLAR PARA EL**

**PARTICIPANTE**

*Buenos días/tardes,*

*Mi nombre es XXXXX y me presento en nombre de la FCIEN y de Madrid Salud, un Organismo Autónomo del Ayuntamiento de Madrid. Estamos realizando una encuesta en las personas de 90 años y más para conocer mejor los problemas de salud a esa edad e identificar las causas de la longevidad y del envejecimiento saludable. Esta encuesta consiste en una serie de preguntas que le voy a formular acerca de sus problemas de salud, estilos de vida y otras cuestiones relacionadas. Si le resulta difícil responder a alguna pregunta puede ayudarle a contestar algún miembro de su familia o allegado próximo. El cuestionario no le entretendrá más de 30 minutos. Toda la información que usted nos facilite está sujeta a la legislación actual en materia de confidencialidad y protección de datos y será tratada de forma anónima.*

*¿Tiene alguna duda?*

*¿Desea participar en la encuesta?*

**REALIZACIÓN DE LA ENTREVISTA**

**SI** □ **NO** □

###### Si la entrevista no se puede realizar, indicar el motivo:

No quiere participar………………………….…………..………..…. 1

Demasiado enfermo físicamente………………………………… 2

Deterioro cognitivo severo…………………………………………. 3

Defectos sensoriales o motores………………………………….. 4

Fallecido……………………………………………………………………… 5

Otros…………………………………………………………………………… 6

(*especificar ____________________*_________)

**Fecha de la encuesta** _____/_____/___________

*Notas:*

1. *Es deseable que la encuesta sea respondida en su totalidad por los participantes. No obstante, para que la encuesta sea considerada válida será necesario que al menos se hayan respondido a todas las preguntas marcadas con un asterisco (*)*
2. *La aplicación del apartado C en su conjunto, marcado con dos asteriscos (**), será siempre de obligado cumplimiento excepto si el participante presenta deterioro cognitivo severo (por ejemplo, una demencia tipo Alzheimer) o graves defectos sensoriales (por ejemplo, ceguera), en cuyo caso su aplicación dependerá del estado del participante.*
3. *Si un participante necesita ayuda para responder a algún ítem de la encuesta, este podrá ser cumplimentado por un familiar o cuidador siempre que aparezca también en el cuestionario del ACOMPAÑANTE. En todo caso, la respuesta del proxy se registrará únicamente su cuestionario específico.*

**A. VARIABLES DEMOGRÁFICAS**

* A1. FECHA DE NACIMIENTO Y EDAD

**¿Cuál es la fecha de su nacimiento? Si no la sabe o duda, ¿cuál es su edad?**

A1.1. Fecha de nacimiento _____/_____/___________

A1.2. Edad _______

* A2. SEXO

Hombre………………………………………………….………………..…. 1

Mujer……..…………………….…………………………….………………. 2

* A3. NIVEL DE ESTUDIOS

**¿Cuál es su nivel de estudios?**

No sabe leer ni escribir………….……………...……………………. 1

Sin estudios………………………….……………………………..………. 2

Estudios Primarios…………………………………………..………….. 3

Bachiller / Estudios Secundarios………………...............…… 4

Estudios Superiores………………………………………………..…… 5

No sabe……………………………………………………………………….. 8

No contesta…………………………………………………………………. 9

* A4. CATEGORÍA PROFESIONAL

**¿Cuál ha sido su categoría profesional principal?**

TRABAJADOR AUTÓNOMO O EMPRESARIO POR CUENTA PROPIA

Sin asalariados…………………………………………..……………….. 1

Con menos de 10 asalariados……………………………………… 2

Con 10 o más asalariados……………………………………………. 3

TRABAJADOR POR CUENTA AJENA

Gerente de una empresa con 10 o más empleados……. 4

Gerente de una empresa con menos de 10 empleados 5

Capataz, supervisor o encargado………………………………… 6

Otro asalariado………………………………………………..……….… 7

SIN TRABAJO REMUNERADO

Ama de casa………………………………………………..……….……… 8

Otro…………..………………………………………………..……….……… 9

No sabe……………………………………………………………………….. 98

No contesta…………………………………………………………………. 99

* A5. TIPO DE OCUPACIÓN

**¿Cuál ha sido su ocupación laboral principal?**

Gerencia / Dirección de empresas………………………..……. 1

Profesional………………………………………………………………….. 2

Trabajador administrativo…………………………………………… 3

Trabajador manual cualificado……………………………………. 4

Trabajador manual no cualificado………………………………. 5

No sabe……………………………………………………………………….. 8

No contesta…………………………………………………………………. 9

* A6. ESTADO CIVIL

**¿Cuál es su estado civil?**

Casado, en pareja………………………………………………………… 1

Soltero…………………………………………………………….....………. 2

Separado, Divorciado………..………………………………………… 3

Viudo……………………………………………………………………..……. 4

No sabe……………………………………………………………………….. 8

No contesta…………………………………………………………………. 9

* A7. CONVIVENCIA

**¿Con quién vive?**

Solo……………………………………………………………………..………. 1

En pareja……………………………………………………………………… 2

En pareja con hijos………………………………………………………. 3

Con hijos……………………………………………………………………… 4

Con otros familiares……………………………………………………. 5

Con cuidadores profesionales……………………………………… 6

Otro……………………………………………………………………..……… 7

A7.1. (*especificar _____________*_________)

A8. FAMILIA / RED SOCIAL

**Número de personas con los que mantiene contacto al menos una vez por semana**

A8.1. Hijos ________

A8.2. Hermanos ________

A8.3. Sobrinos ________

A8.4. Amigos ________

A8.5. Otros ________

A8.6. (*especificar _____________*_________)

A9. LUGAR DE NACIMIENTO

**¿Dónde nació?**

Municipio de Madrid…………………………………………………… 1

Otro municipio de España…………………………………………… 2

A9.1. (*especificar* ___________________________)

A9.2. ¿Desde cuándo reside en la ciudad de Madrid? ______ años

Extranjero……………………………………………………………………. 3

A9.3. (*especificar* ___________________________)

A9.4. ¿Desde cuándo reside en la ciudad de Madrid? ______ años

A10. CARACTERÍSTICAS DE LA VIVIENDA

A10.1. Régimen de tenencia de la vivienda principal

En propiedad del núcleo familiar……..……………... 1

En alquiler………………………………………..……………… 2

Otro 3

A10.2. (*especificar* ___________________________)

A10.3. ¿Su edificio tiene ascensor?

Sí…………………………………………..…….…..……………... 1

No………………………………………..…….…..……………... 2

A10.4. ¿Cuántas estancias de 4m² o más tiene su vivienda incluyendo dormitorios, comedores, salones, cuartos de estar y cocinas? ________

**B. MORBILIDAD Y LIMITACIÓN DE LA ACTIVIDAD**

* B1. PATOLOGÍAS

**A continuación le voy a leer una lista con una serie de enfermedades o problemas de salud. En cada una de ellas dígame si su médico le ha dicho que la padece o no.**

|  | **SÍ** | **NO** | **NS** | **NC** |
| --- | --- | --- | --- | --- |
| B1.1. Hipertensión arterial (tensión elevada)…………………………….….……… | 1 | 2 | 8 | 9 |
| B1.2. Colesterol alto……………………………………………………………………….…… | 1 | 2 | 8 | 9 |
| B1.3. Diabetes (azúcar elevada)…………………………………………………….……. | 1 | 2 | 8 | 9 |
| B1.4. Angina de pecho / infarto…………………………………………………….……. | 1 | 2 | 8 | 9 |
| B1.5. Otras enfermedades del corazón………………………………………….……. | 1 | 2 | 8 | 9 |
| B1.6. Bronquitis crónica, enfisema pulmonar, EPOC………………………….. | 1 | 2 | 8 | 9 |
| B1.7. Artrosis, artritis o reumatismos……………………………………………….…… | 1 | 2 | 8 | 9 |
| B1.8. Osteoporosis…………………………………………………………………….….…….. | 1 | 2 | 8 | 9 |
| B1.9. Depresión………………………………………………………………………….……….. | 1 | 2 | 8 | 9 |
| B1.10. Ansiedad…………………………………………………………………………….……. | 1 | 2 | 8 | 9 |
| B1.11. Accidente cerebrovascular, ictus, embolia cerebral……………….… | 1 | 2 | 8 | 9 |
| B1.12. Enfermedad de Alzheimer…………………………………………………..……….. | 1 | 2 | 8 | 9 |
| B1.13. Enfermedad de Parkinson……………………………………….……………….. | 1 | 2 | 8 | 9 |
| B1.14. Demencia senil u otro tipo de demencia……………………………….... | 1 | 2 | 8 | 9 |
| B1.15. Cáncer o tumor…………………………………………………………………………  (*especificar* ______________________________) | 1 | 2 | 8 | 9 |

B2. MEDICACIÓN

**¿Qué medicamentos diferentes toma a diario (gotas, pastillas, inyecciones, supositorios, pomadas, parches, etc.)?**

| *(anotar uno por uno todos los medicamentos que tome el participante)* |
| --- |
| B2.1. ___________________________________________________ |
| B2.2. ___________________________________________________ |
| B2.3. ___________________________________________________ |
| B2.4. ___________________________________________________ |
| B2.5. ___________________________________________________ |
| B2.6. ___________________________________________________ |
| B2.7. ___________________________________________________ |
| B2.8. ___________________________________________________ |
| B2.9. ___________________________________________________ |
| B2.10. __________________________________________________ |
| B2.11. __________________________________________________ |
| B2.12. __________________________________________________ |

**** C. ESTADO COGNITIVO (TICSM-SP)**

** C1. DETERIORO COGNITIVO SUBJETIVO

|  | **SÍ** | **NO** | **NS** | **NC** |
| --- | --- | --- | --- | --- |
| C1.1. ¿Tiene usted problemas de memoria?.......................................... | 1 | 2 | 8 | 9 |
| *Preguntar solo a los que han respondido de modo afirmativo:* |  |  |  |  |
| C1.2. ¿Desde hace cuánto tiempo? ___________ años |  |  |  |  |
| C1.3. ¿Tiene usted problemas de atención o concentración?.............. | 1 | 2 | 8 | 9 |
| C1.4. ¿Le preocupan estos problemas de atención o de memoria?..... | 1 | 2 | 8 | 9 |
| C1.5. ¿Ha consultado usted a su médico por este motivo?.................... | 1 | 2 | 8 | 9 |
| C1.6. ¿Le afectan en su vida cotidiana?................................................. | 1 | 2 | 8 | 9 |
| C1.7. En relación a su memoria, se encuentra usted peor que  otras personas de su misma edad?.......................................................... | 1 | 2 | 8 | 9 |

** C2. TELEPHONE INTERVIEW FOR COGNITIVE STATUS

| **Ítem** | | | | **Puntuación** | | | | | | | |
| --- | --- | --- | --- | --- | --- | --- | --- | --- | --- | --- | --- |
|  |  | | |  | | | | | | | |
|  | Por favor, dígame su nombre y apellidos: | | |  | | | | | | | |
| C2.1. | Nombre: | | | 0 | | | | 1 | | | |
| C2.2. | Apellidos: | | | 0 | | | | 1 | | | |
| C2.3. | ¿En qué año estamos? | | | 0 | | | | 1 | | | |
| C2.4. | ¿En qué estación del año estamos? | | | 0 | | | | 1 | | | |
| C2.5. | ¿En qué mes estamos? | | | 0 | | | | 1 | | | |
| C2.6. | ¿Qué día del mes es hoy? | | | 0 | | | | 1 | | | |
| C2.7. | ¿Qué día de la semana es hoy? | | | 0 | | | | 1 | | | |
|  | ¿Cuál es su dirección de correo postal? | | |  | | | | | | | |
| C2.8. | País: | | | 0 | | | | 1 | | | |
| C2.9. | Localidad: | | | 0 | | | | 1 | | | |
| C2.10. | Calle: | | | 0 | | | | 1 | | | |
| C2.11. | Número: | | | 0 | | | | 1 | | | |
| C2.12. | Código Postal: | | | 0 | | | | 1 | | | |
|  |  | | |  | | | | | | | |
|  |  | | |  | | | | | | | |
| C2.13. | Cuente hacia atrás, de uno en uno, desde 20 hasta 1: | | |  | | | | | | | |
|  | Intento 1: | | |  | | | | | | | |
|  | Si falla en el intento 1, Intento 2: | | | 0 | | | 1 | | | 2 | |
|  | [Si el participante cuenta correctamente en el intento 1, se le otorga una puntuación de 2. Por el contrario, si comete algún error se le pedirá que lo vuelva a intentar por segunda vez; si realiza la prueba correctamente en el intento 2 se le otorgará una puntuación de 1. Si no es capaz de completar la prueba en ninguno de los dos intentos se le dará una puntuación de 0] | | |  | | |  | | |  | |
|  |  | | |  | | | | | | | |
|  |  | | |  | | | | | | | |
|  | A continuación, voy a leerle una lista de 10 palabras. Por favor, escuche con atención e intente retenerlas. Cuando yo termine tendrá que decirme todas las palabras que pueda recordar en cualquier orden. ¿Preparado? Las palabras son: CABINA, PIPA, ELEFANTE, PECHO, SEDA, TEATRO, RELOJ, LÁTIGO, ALMOHADA, GIGANTE. Ahora dígame cuáles recuerda: | | |  | | | | | | | |
| C2.14. | CABINA | | | 0 | | | | 1 | | | |
| C2.15. | PIPA | | | 0 | | | | 1 | | | |
| C2.16. | ELEFANTE | | | 0 | | | | 1 | | | |
| C2.17. | PECHO | | | 0 | | | | 1 | | | |
| C2.18. | SEDA | | | 0 | | | | 1 | | | |
| C2.19. | TEATRO | | | 0 | | | | 1 | | | |
| C2.20. | RELOJ | | | 0 | | | | 1 | | | |
| C2.21. | LÁTIGO | | | 0 | | | | 1 | | | |
| C2.22. | ALMOHADA | | | 0 | | | | 1 | | | |
| C2.23. | GIGANTE | | | 0 | | | | 1 | | | |
|  | [Se otorga un punto por cada palabra recordada. Las formas en plural son consideradas correctas. No se penalizan las repeticiones ni las intrusiones]. | | |  | | | |  | | | |
|  |  | | |  | | | | | | | |
|  |  | | |  | | | | | | | |
|  | Ahora quiero que reste de 7 en 7 empezando en 100. Es decir, ¿cuánto es 100 menos 7? Continúe restando: | | |  | | | | | | | |
| C2.24. | 93 | | | 0 | | | | 1 | | | |
| C2.25. | 86 | | | 0 | | | | 1 | | | |
| C2.26. | 79 | | | 0 | | | | 1 | | | |
| C2.27. | 72 | | | 0 | | | | 1 | | | |
| C2.28. | 65 | | | 0 | | | | 1 | | | |
|  | [Se considera una respuesta correcta siempre que la resta con respecto al número anterior sea igual a la sustracción de 7, con independencia de que la respuesta dada fuera o no correcta] | | |  | | | |  | | | |
|  |  | | |  | | | | | | | |
|  |  | | |  | | | | | | | |
| C2.29. | ¿Qué utensilio se emplea para cortar el papel? | | | 0 | | | | 1 | | | |
|  | [Solo se consideran válidas las respuestas “tijeras” o “cúter”] | | |  | | | |  | | | |
|  |  | | |  | | | | | | | |
|  |  | | |  | | | | | | | |
| C2.30. | ¿Cuántas cosas hay en una docena? | | | 0 | | | | 1 | | | |
|  | [Solo se considera válida la respuesta “12”] | | |  | | | |  | | | |
|  |  | | |  | | | | | | | |
|  |  | | |  | | | | | | | |
| C2.31. | ¿Cómo se llama a la planta verde con pinchos que vive en el desierto? | | | 0 | | | | 1 | | | |
|  | [Solo se considera válida la respuesta “cactus”] | | |  | | | |  | | | |
|  |  | | |  | | | | | | | |
|  |  | | |  | | | | | | | |
| C2.32. | ¿De qué animal viene la lana? | | | 0 | | | | 1 | | | |
|  | [Solo se consideran válidas las respuestas “oveja” o “cordero”] | | |  | | | |  | | | |
|  |  | | |  | | | | | | | |
|  |  | | |  | | | | | | | |
| C2.33. | Repita la siguiente frase: “NI SÍ, NI NO, NI PERO” | | | 0 | | | | 1 | | | |
|  | [Se otorga un punto solo si la repetición es correcta en el primer intento] | | |  | | | |  | | | |
|  |  | | |  | | | | | | | |
|  |  | | |  | | | | | | | |
| C2.34. | Repita la siguiente frase: “METODISTA EPISCOPAL” | | | 0 | | | | 1 | | | |
|  | [Se otorga un punto solo si la repetición es correcta en el primer intento] | | |  | | | |  | | | |
|  |  | | |  | | | | | | | |
|  |  | | |  | | | | | | | |
| C2.35. | ¿Me puede decir el nombre del Presidente del Gobierno? | | | 0 | | | | 1 | | | |
|  | [Se otorga un punto si recuerda correctamente el nombre y/o el apellido] | | |  | | | |  | | | |
|  |  | | |  | | | | | | | |
|  |  | | |  | | | | | | | |
| C2.36. | ¿Y quién es el Rey de España? | | | 0 | | | | 1 | | | |
|  | [Se otorga un punto solo si dice “Felipe VI”] | | |  | | | |  | | | |
|  |  | | |  | | | | | | | |
|  |  | | |  | | | | | | | |
| C2.37. | Por favor, golpee 5 veces su teléfono con uno de sus dedos: | | | 0 | | | | 1 | | | |
|  | [Se otorgan dos puntos si se escuchan exactamente 5 golpeteos y un punto si el individuo golpea un número distinto de veces] | | |  | | | |  | | | |
|  |  | | |  | | | | | | | |
|  |  | | |  | | | | | | | |
| C2.38. | Le voy a decir una palabra y quiero que me dé su contrario. Por ejemplo, si le digo calor, lo contrario sería frío. ¿Qué es lo opuesto a Occidente? | | | 0 | | | | 1 | | | |
|  | [Solo se consideran válidas las respuestas “Oriente” o “Este”] | | |  | | | |  | | | |
|  |  | | |  | | | | | | | |
|  |  | | |  | | | | | | | |
| C2.39. | ¿Y qué es lo contrario a generoso? | | | 0 | | | | 1 | | | |
|  | [Se consideran válidas las respuestas “tacaño”, “roñoso”, “egoísta” o similares] | | |  | | | |  | | | |
|  |  | | |  | | | | | | | |
|  |  | | |  | | | | | | | |
|  | Antes le leí una lista de 10 palabras. ¿Podría decirme todas las que recuerde? | | |  | | | | | | | |
| C2.40. | CABINA | | | 0 | | | | 1 | | | |
| C2.41. | PIPA | | | 0 | | | | 1 | | | |
| C2.42. | ELEFANTE | | | 0 | | | | 1 | | | |
| C2.43. | PECHO | | | 0 | | | | 1 | | | |
| C2.44. | SEDA | | | 0 | | | | 1 | | | |
| C2.45. | TEATRO | | | 0 | | | | 1 | | | |
| C2.46. | RELOJ | | | 0 | | | | 1 | | | |
| C2.47. | LÁTIGO | | | 0 | | | | 1 | | | |
| C2.48. | ALMOHADA | | | 0 | | | | 1 | | | |
| C2.49. | GIGANTE | | | 0 | | | | 1 | | | |
|  | [Se otorga un punto por cada palabra recordada. Las formas en plural son consideradas correctas. No se penalizan las repeticiones ni las intrusiones] | | |  | | | |  | | | |
|  |  | | |  | | | |  | | | |
|  |  | | |  | | | |  | | | |
| C2.50. | Deletree al revés la palabra MUNDO | 0 | 1 | | 2 | 3 | | | 4 | | 5 |
|  |  | | |  | | | |  | | | |

**D. SALUD PERCIBIDA Y CALIDAD DE VIDA**

D1. SALUD AUTOPERCIBIDA

**En general, ¿cómo calificaría su estado de salud en los últimos 12 meses?**

Muy bueno………………………………………………………………….. 1

Bueno……………………………………………………………………..…... 2

Regular……………………………………………………………………..…. 3

Malo……………………………………………………………………..…….. 4

Muy malo……………………………………………………………………. 5

No sabe……………………………………………………………………….. 8

No contesta…………………………………………………………………. 9

D2. TERMÓMETRO DE SALUD (*mostrar imagen del Anexo I*)

**En una escala de 0 a 100, donde 0 representa el peor estado de salud que pueda imaginar y 100 el mejor estado de salud que pueda imaginar, indique cómo cree que se encuentra su estado de salud en el día de hoy _________**

D3. FORMA FÍSICA EN LAS ÚLTIMAS DOS SEMANAS

**Durante las dos últimas semanas, ¿cuál ha sido la máxima actividad física que pudo realizar durante al menos dos minutos?**

Muy intensa (p. ej., correr deprisa)................................. 1

Intensa (p. ej., correr con suavidad)................................ 2

Moderada (p. ej., caminar a paso rápido)....................... 3

Ligera (p. ej., caminar despacio)...................................... 4

Muy ligera (p. ej., caminar muy lentamente).................. 5

Ninguna (p. ej., no poder caminar)……………………………… 6

D4. SENTIMIENTOS EN LAS ÚLTIMAS DOS SEMANAS

**Durante las dos últimas semanas, ¿en qué medida le han molestado los problemas emocionales tales como sentimientos de ansiedad, depresión, irritabilidad o tristeza y desánimo?**

Nada en absoluto............................................................ 1

Un poco........................................................................... 2

Moderadamente............................................................. 3

Bastante........................................................................... 4

Intensamente.................................................................. 5

D5. ACTIVIDADES COTIDIANAS EN LAS ÚLTIMAS DOS SEMANAS

**Durante las dos últimas semanas, ¿cuánta dificultad ha tenido al hacer sus actividades o tareas habituales tanto dentro como fuera de casa, a causa de su salud física o por problemas emocionales?**

Nada en absoluto............................................................ 1

Un poco de dificultad...................................................... 2

Dificultad moderada........................................................ 3

Mucha dificultad.............................................................. 4

Todo, no he podido hacer nada....................................... 5

D6. ACTIVIDADES SOCIALES EN LAS ÚLTIMAS DOS SEMANAS

**Durante las dos últimas semanas, ¿su salud física y estado emocional han limitado sus actividades sociales con la familia, amigos, vecinos o grupos?**

No, nada en absoluto...................................................... 1

Ligeramente..................................................................... 2

Moderadamente............................................................. 3

Bastante........................................................................... 4

Muchísimo....................................................................... 5

D7. CAMBIO EN EL ESTADO DE SALUD EN LAS ÚLTIMAS DOS SEMANAS

**¿Cómo calificaría ahora su estado de salud, en comparación con el de hace dos semanas?**

Mucho mejor................................................................... 1

Un poco mejor................................................................. 2

Igual, por el estilo............................................................ 3

Un poco peor................................................................... 4

Mucho peor..................................................................... 5

D8. ESTADO DE SALUD EN LAS ÚLTIMAS DOS SEMANAS

**Durante las dos últimas semanas, ¿cómo calificaría su salud general?**

Excelente......................................................................... 1

Muy buena...................................................................... 2

Buena............................................................................... 3

Regular............................................................................ 4

Mala................................................................................. 5

D9. DOLOR EN LAS ÚLTIMAS DOS SEMANAS

**Durante las dos últimas semanas, ¿cuánto dolor ha tenido?**

Nada de dolor.................................................................. 1

Dolor muy leve................................................................ 2

Dolor ligero...................................................................... 3

Dolor moderado.............................................................. 4

Dolor intenso................................................................... 5

D10. APOYO SOCIAL EN LAS ÚLTIMAS DOS SEMANAS

**Durante las dos últimas semanas, ¿había alguien dispuesto a ayudarle si hubiera necesitado ayuda? Por ejemplo: *se encontraba nervioso, solo o triste; *caía enfermo y tenía que quedarse en la cama; *necesitaba hablar con alguien; *necesitaba ayuda con las tareas de la casa; *necesitaba ayuda para cuidar de sí mismo.**

Sí, todo el mundo estaba dispuesto a ayudarme........... 1

Sí, bastante gente............................................................ 2

Sí, algunas personas........................................................ 3

Sí, alguien había............................................................... 4

Nadie en absoluto........................................................... 5

D11. CALIDAD DE VIDA EN LAS ÚLTIMAS DOS SEMANAS

**¿Qué tal le han ido las cosas en las dos últimas semanas?**

Estupendamente, no podían ir mejor............................. 1

Bastante bien................................................................... 2

A veces bien, a veces mal. Bien y mal a partes iguales... 3

Bastante mal.................................................................... 4

Muy mal, no podían haber ido peor................................ 5

D12. SOLEDAD

**¿Con qué frecuencia se ha sentido usted solo/a durante el último año?**

Siempre o casi siempre.................................................... 1

Bastantes veces............................................................... 2

Pocas veces...................................................................... 3

Nunca o casi nunca.......................................................... 4

No sabe……………………………………………………………………….. 8

No contesta…………………………………………………………………. 9

**E. DEPENDENCIA FUNCIONAL**

E1. DISCAPACIDAD

**¿Tiene reconocida una discapacidad/minusvalía?**

Sí…………………………………………………………………………………. 1

No……………………………………………………………………………….. 2

No sabe……………………………………………………………………….. 8

No contesta…………………………………………………………………. 9

E2. PORCENTAJE DE DISCAPACIDAD

**Si lo sabe, díganos qué grado de discapacidad tiene reconocido**: _______ %

E3. DEPENDENCIA

**¿Ha solicitado la valoración de su situación de dependencia?**

Sí, y tengo el dictamen de la Comunidad de Madrid…... 1

Sí, pero estoy pendiente de la resolución final…………... 2

No……………………………………………………………………………….. 3

No sabe……………………………………………………………………….. 8

No contesta…………………………………………………………………. 9

E4. GRADO DE DEPENDENCIA RECONOCIDO

**Si lo sabe, díganos qué grado de dependencia tiene reconocido**: _______

* E5. DIFICULTADES SENSORIALES VISUALES

**¿Puede leer con normalidad el periódico o ver la televisión (con gafas o lentillas si las necesita)?**

Sí, sin dificultad................................................................ 1

Sí, con alguna dificultad................................................... 2

E5.1. (*especificar el motivo*___________________________)

Sí, con mucha dificultad................................................... 3

E5.2. (*especificar el motivo*___________________________)

No, no puede hacerlo...................................................... 4

* E6. DIFICULTADES SENSORIALES AUDITIVAS

**¿Puede oír con normalidad lo que se dice en una conversación de tres o cuatro personas (con audífono si lo necesita)?**

Sí, sin dificultad................................................................ 1

Sí, con alguna dificultad................................................... 2

E6.1. (*especificar el motivo*___________________________)

Sí, con mucha dificultad................................................... 3

E6.2. (*especificar el motivo*___________________________)

No, no puede hacerlo...................................................... 4

* E7. ÍNDICE DE KATZ

**Ahora voy a hacerle algunas preguntas sobre actividades corrientes de la vida de cualquier persona. Dígame si usted es capaz de...**

| E7.1. BAÑARSE (CON ESPONJA, DUCHA O BAÑERA) |  |
| --- | --- |
| Independiente. Necesita ayuda para lavarse una sola parte (como la espalda o una extremidad incapacitada) o se baña completamente sin ayuda……………….. | 1 |
| Dependiente. Necesita ayuda para lavarse más de una parte del cuerpo; necesita ayuda para salir o entrar en la bañera o no se lava solo………….………… | 0 |
| E7.2. VESTIRSE |  |
| Independiente. Coge la ropa de armarios y cajones, se pone la ropa, se pone adornos y abrigos; utiliza cremalleras, se excluye el atarse los zapatos…………… | 1 |
| Dependiente: No se viste solo o permanece vestido parcialmente…………………. | 0 |
| E7.3. USAR EL RETRETE |  |
| Independiente. Accede al retrete, entra y sale en él; se arregla la ropa; se limpia los órganos excretores (puede utilizar o no soportes mecánicos)…………. | 1 |
| Dependiente. Usa orinal o cuña o precisa ayuda para acceder y utilizar el retrete…………………………………………………………………………………………………………… | 0 |
| E7.4. MOVILIDAD |  |
| Independiente. Entra y sale de la cama y se sienta y levanta de la silla independientemente (puede usar o no soportes mecánicos)………………………….. | 1 |
| Dependiente. Precisa ayuda para utilizar la cama y/o la silla; no realiza uno o más desplazamientos……………………………………………………………………………………… | 0 |
| E7.5. CONTINENCIA |  |
| Independiente. Control completo de micción y defecación…………………………….. | 1 |
| Dependiente. Incontinencia urinaria o fecal parcial o total; control total o parcial mediante enemas, sondas, o el uso reglado de orinales y/o cuñas……… | 0 |
| E7.6. ALIMENTACIÓN |  |
| Independiente. Lleva la comida del plato o su equivalente a la boca (se excluye cortar carne, preparar la comida, untar mantequilla en pan, etc.)……… | 1 |
| Dependiente. Precisa ayuda para el acto de alimentarse (véase arriba); no come en absoluto o nutrición parenteral………………………………………………………… | 0 |

**F. HÁBITOS Y ESTILOS DE VIDA**

F1. PESO

**Aproximadamente, ¿cuánto pesa sin zapatos ni ropa?** _______ kg

F2. ESTATURA

**Aproximadamente, ¿cuánto mide sin zapatos?** _______ cm

F3. HORAS DE SUEÑO AL DÍA

**¿Podría indicarme cuántas horas duerme aproximadamente al día incluyendo las siestas?** _______ horas diarias

F4. CALIDAD DE SUEÑO

**¿Y qué tal duerme? Diría usted que duerme muy bien, bien, regular, mal o muy mal.**

Muy bien…………………………………………………………………….. 1

Bien………………………………………………………………………..…... 2

Regular……………………………………………………………………..…. 3

Mal..……………………………………………………………………..…….. 4

Muy mal………………………………………………………………………. 5

No sabe……………………………………………………………………….. 8

No contesta…………………………………………………………………. 9

F5. CONSUMO DE TABACO

**¿Podría decirme si fuma actualmente?**

Sí, diariamente................................................................. 1

F5.1. (*especificar, ¿cuántos años lleva fumando?*_________)

Sí, pero no diariamente………………………………………………. 2

F5.2. (*especificar, ¿cuántos años lleva fumando?*_________)

No actualmente, pero ha fumado antes……………………… 3

F5.3. (*especificar, ¿durante cuántos años fumó?*_________)

F5.4. (*especificar, ¿cuántos años hace que lo dejó? _________*)

No fuma, ni ha fumado nunca de manera habitual…….. 4

No sabe……………………………………………………………………….. 8

No contesta…………………………………………………………………. 9

F6. CONSUMO DE ALCOHOL

**¿Podría decirme si bebe algo de alcohol actualmente?**

Sí, al menos cinco veces por semana............................ 1

F6.1. (*especificar, ¿qué tipo de bebida alcohólica?*_________)

F6.2. (*especificar, ¿qué cantidad de bebida alcohólica?*_________)

F6.3. (*especificar, ¿desde hace cuántos años?*_________)

No actualmente, pero he bebido antes………………..……… 2

F6.4. (*especificar, ¿durante cuántos años bebió?*_________)

F6.5. (*especificar, ¿cuántos años hace que lo dejó? _________*)

No bebe, ni ha bebido nunca de manera habitual……….. 3

No sabe……………………………………………………………………….. 8

No contesta…………………………………………………………………. 9

F7. ALIMENTACIÓN

**¿Con qué frecuencia consume a la semana los siguientes grupos de alimentos?**

|  | **0 días** | **1-2 días** | **3 días** | **4-6 días** | **A diario** |
| --- | --- | --- | --- | --- | --- |
| F7.1. Verduras, ensaladas y hortalizas…………………….…....... | 0 | 1 | 2 | 3 | 4 |
| F7.2. Fruta fresca (excluyendo zumos naturales)…………….. | 0 | 1 | 2 | 3 | 4 |
| F7.3. Aceite de oliva virgen extra…………………………………..…… | 0 | 1 | 2 | 3 | 4 |
| F7.4. Legumbres…………………………………………………………….. | 0 | 1 | 2 | 3 | 4 |
| F7.5. Pescado azul……………………………………………………………. | 0 | 1 | 2 | 3 | 4 |
| F7.6. Frutos secos…………………………………………………………… | 0 | 1 | 2 | 3 | 4 |
| F7.7. Café (con o sin cafeína)…………………………………………… | 0 | 1 | 2 | 3 | 4 |

F8. ACTIVIDADES COTIDIANAS

**¿Con qué frecuencia realiza las siguientes actividades de ocio a la semana?**

|  | **0 días** | **1-2 días** | **3 días** | **4-6 días** | **A diario** |
| --- | --- | --- | --- | --- | --- |
| F8.1. Salir a caminar más de 10 minutos seguidos………….… | 0 | 1 | 2 | 3 | 4 |
| F8.2. Otra actividad física (gimnasia, taichí, etc.)…………..…  (*especificar* __________________________) | 0 | 1 | 2 | 3 | 4 |
| F8.3. Actividades creativas (pintar, escribir, etc.)…………….. | 0 | 1 | 2 | 3 | 4 |
| F8.4. Salir con amigos a pasear, tomar un café, etc………..… | 0 | 1 | 2 | 3 | 4 |
| F8.5. Hacer pasatiempos (crucigramas, sudokus, etc.)…….. | 0 | 1 | 2 | 3 | 4 |
| F8.6. Asistir a cursos o talleres en centros de mayores……. | 0 | 1 | 2 | 3 | 4 |
| F8.7. Ir al cine, teatro, conciertos, etc……………………………… | 0 | 1 | 2 | 3 | 4 |
| F8.8. Escuchar música……………………………………………………… | 0 | 1 | 2 | 3 | 4 |
| F8.9. Ver la televisión; oír la radio………………………………….… | 0 | 1 | 2 | 3 | 4 |
| F8.10. Leer (periódico, libros o revistas)……………………………. | 0 | 1 | 2 | 3 | 4 |
| F8.11. Utilizar nuevas tecnologías (móvil, ordenador)….…. | 0 | 1 | 2 | 3 | 4 |

**ANEXO I**

El mejor estado de salud imaginable

**TERMÓMETRO DE SALUD**

Para ayudar a la gente a describir lo bueno o malo que es su estado de salud hemos dibujado una escala parecida a un termómetro en el cual se marca con un 100 el mejor estado de salud que pueda imaginarse y con un 0 el peor estado de salud que pueda imaginarse.

Nos gustaría que nos indicara en esta escala, en su opinión, lo bueno o malo que es su estado de salud en el día de HOY. Por favor, dibuje una línea desde el casillero donde dice "Su estado de salud de hoy" hasta el punto del termómetro que en su opinión indique lo bueno o malo que es su estado de salud en el día de HOY.

**Su estado de salud HOY**

El peor estado de salud imaginable
