# Supplementary material for: MADRID+90 study on factors associated with longevity: Study design and preliminary data
Source: PLoS One. 2021 May 17;16(5):e0251796. doi: 10.1371/journal.pone.0251796 (PMC8128242; doi:10.1371/journal.pone.0251796)
Supplement: S2 File — (DOCX) [file pone.0251796.s002.docx]

**SURVEY QUESTIONNAIRE “RESEARCH PROGRAM IN LONGEVITY SPAIN-PORTUGAL +90” (PILEP+90)**

**COPY FOR THE**

**PARTICIPANT**

*Good morning/afternoon,*

*My name is XXXXX and I am introducing myself on behalf of FCIEN and Madrid Salud, an Autonomous Organization of the Madrid City Council. We are conducting a survey of people aged 90 and over to learn more about health problems at that age and to identify the causes of longevity and healthy aging. This survey consists of a series of questions that I am going to ask you about your health problems, lifestyles and other related issues. If you find it difficult to answer any of the questions, a family member or close relative may be able to help you answer. The questionnaire should not take more than 30 minutes. All information you provide is subject to current legislation on confidentiality and data protection and will be treated anonymously.*

*Do you have any questions?*

*Would you like to participate in the survey?*

**CONDUCTING THE INTERVIEW**

**YES** □ **NO** □

###### If the interview cannot take place, indicate the reason why:

Does not want to participate..………….…………..………..…. 1

Too physically ill……………………….………………………………… 2

Severe cognitive impairment..……………………………………. 3

Sensory or motor defects………..………………………………….. 4

Deceased..……………………………………………………………..…… 5

Others…………………………………………………………………….…… 6

(*specify ____________________*_________)

**Survey date** _____/_____/___________

*Notes:*

1. *It is desirable that the survey be answered in its entirety by the participants. However, for the survey to be considered valid, at least all questions marked with an asterisk (*) must be answered.*
2. *The application of section C as a whole, marked with two asterisks (**), will always be mandatory unless the participant has severe cognitive impairment (e.g., Alzheimer's dementia) or severe sensory defects (e.g., blindness), in which case its application will depend on the participant's condition.*
3. *If a participant needs help in answering any item of the survey, it may be completed by a family member or caregiver as long as it also appears in the COMPANION questionnaire. In any case, the proxy's response will be recorded only on his or her specific questionnaire.*

**A. DEMOGRAPHIC VARIABLES**

* A1. DATE OF BIRTH AND AGE

**What is your date of birth? If you don't know or doubt it, what is your age?**

A1.1. Date of birth _____/_____/___________

A1.2. Age _______

* A2. GENDER

Male……..……………………………………………….………………..…. 1

Female…..…………………….…………………………….………………. 2

* A3. EDUCATIONAL ATTAINMENT

**What is your level of education?**

Cannot read or write….………….……………...……………………. 1

No education……………………….……………………………..………. 2

Primary Education…………………………………………..………….. 3

Secundary Education……………….………………...............…… 4

Higher Education….………………………………………………..…… 5

Does not know…………………………………………………………….. 8

Does not answer…………………………………………………………. 9

* A4. PROFESSIONAL CATEGORY

**What has been your main professional category?**

SELF-EMPLOYED OR ENTREPENEUR

Without employees…………………………………..……………….. 1

With less than 10 employees.……………………………………… 2

With 10 or more employees.………………………………………. 3

EMPLOYED PERSON

Manager of a company with 10 or more employees..…. 4

Manager of a company with fewer than10 employees.. 5

Foreman, supervisor or manager………………………………… 6

Other employee..……………………………………………..……….… 7

WITHOUT PAID WORK

Housekeeper.……………………………………………..……….……… 8

Other………..………………………………………………..……….……… 9

Does not know..………………………………………………………….. 98

Does not answer…………………………………………………………. 99

* A5. TYPE OF OCCUPATION

**What has been your main occupation?**

Management / Business Administration………………..……. 1

Professional.……………………………………………………………….. 2

Clerical worker……………….…………………………………………… 3

Skilled manual worker…………..……………………………………. 4

Unskilled manual worker…………….………………………………. 5

Does not know…………………………………………………………….. 8

Does not answer …………………………………………………………. 9

* A6. MARITAL STATUS

**What is your marital status?**

Married, in couple.……………………………………………………… 1

Single..…………………………………………………………….....………. 2

Separated, Divorced..………..………………………………………… 3

Widower………………………………………………………………..……. 4

Does not know…………………………………………………………….. 8

Does not answer …………………………………………………………. 9

* A7. COEXISTENCE

**Who do you live with?**

Alone…………………………………………………………………..………. 1

As a couple..………………………………………………………………… 2

As a couple with children.……………………………………………. 3

With children….…………………………………………………………… 4

With other family members..………………………………………. 5

With caregivers…………………….……………………………………… 6

Other situation.....………………………………………………..……… 7

A7.1. (*especify _____________*_________)

A8. FAMILY / SOCIAL NETWORK

**Number of people with whom you have contact at least once a week**

A8.1. Children ________

A8.2. Siblings ________

A8.3. Nephews ________

A8.4. Friends ________

A8.5. Others ________

A8.6. (*especify _____________*_________)

A9. PLACE OF BIRTH

**Where were you born?**

City of Madrid………..…………………………………………………… 1

Other city in Spain………….…………………………………………… 2

A9.1. (*especify* ___________________________)

A9.2. How long have you lived in the city of Madrid? ______ years

Abroad…..……………………………………………………………………. 3

A9.3. (*especify* ___________________________)

A9.4. How long have you lived in the city of Madrid? ______ years

A10. CHARACTERISTICS OF THE HOUSE

A10.1. Tenancy regime of the main dwelling

Owned by the family unit……….. ……..……………... 1

For rent….………………………………………..……………… 2

Other 3

A10.2. (*especify* ___________________________)

A10.3. Does your building have an elevator?

Yes………………………………………..…….…..……………... 1

No………………………………………..…….…..……………... 2

A10.4. How many rooms of 4m² or more does your home have including bedrooms, dining rooms, living rooms, family rooms and kitchens? ________

**B. MORBIDITY AND ACTIVITY LIMITATION**

* B1. DISEASES

**Next I am going to read you a list with a series of diseases or health problems. For each one, tell me if your doctor has told you that you have it or not.**

|  | **YES** | **NO** | **N/K** | **N/A** |
| --- | --- | --- | --- | --- |
| B1.1. Hypertension (high blood pressure)….…………………………….….……… | 1 | 2 | 8 | 9 |
| B1.2. Cholesterol…..……………………………………………………………………….…… | 1 | 2 | 8 | 9 |
| B1.3. Diabetes……………………………………………………………………………….……. | 1 | 2 | 8 | 9 |
| B1.4. Angina pectoris / heart attack..…………………………………………….……. | 1 | 2 | 8 | 9 |
| B1.5. Other heart diseases………………..………………………………………….……. | 1 | 2 | 8 | 9 |
| B1.6. Chronic bronchitis, pulmonary emphysema, COPD…………………….. | 1 | 2 | 8 | 9 |
| B1.7. Arthrosis, arthritis or rheumatism………………………………………….…… | 1 | 2 | 8 | 9 |
| B1.8. Osteoporosis…………………………………………………………………….….…….. | 1 | 2 | 8 | 9 |
| B1.9. Depression………………………………………………………………………….……….. | 1 | 2 | 8 | 9 |
| B1.10. Anxiety….…………………………………………………………………………….……. | 1 | 2 | 8 | 9 |
| B1.11. Stroke, cerebral embolism……………………………………..……………….… | 1 | 2 | 8 | 9 |
| B1.12. Alzheimer´s disease…….…………………………………………………..……….. | 1 | 2 | 8 | 9 |
| B1.13. Parkinson´s disease………..……………………………………….……………….. | 1 | 2 | 8 | 9 |
| B1.14. Senile dementia or other typE of dementia.………………………….... | 1 | 2 | 8 | 9 |
| B1.15. Cancer or tumor…………………………………………………………………………  (*especify* ______________________________) | 1 | 2 | 8 | 9 |

B2. MEDICATION

**What different medications do you take on a daily basis (drops, pills, injections, suppositories, ointments, patches, etc.)?**

| *(list one by one all medications taken by the participant)* |
| --- |
| B2.1. ___________________________________________________ |
| B2.2. ___________________________________________________ |
| B2.3. ___________________________________________________ |
| B2.4. ___________________________________________________ |
| B2.5. ___________________________________________________ |
| B2.6. ___________________________________________________ |
| B2.7. ___________________________________________________ |
| B2.8. ___________________________________________________ |
| B2.9. ___________________________________________________ |
| B2.10. __________________________________________________ |
| B2.11. __________________________________________________ |
| B2.12. __________________________________________________ |

**** C. COGNITIVE STATUS**

** C1. SUBJECTIVE COGNITIVE DECLINE

|  | **YES** | **NO** | **N/K** | **N/A** |
| --- | --- | --- | --- | --- |
| C1.1. Do you have memory problems?.................................................... | 1 | 2 | 8 | 9 |
| *Ask only those who have answered in the affirmative:* |  |  |  |  |
| C1.2. For how long? ___________ years |  |  |  |  |
| C1.3. Do you have attention or concentration problems?...................... | 1 | 2 | 8 | 9 |
| C1.4. Are you concerned about these attention or memory problems?... | 1 | 2 | 8 | 9 |
| C1.5. ¿ Have you consulted your physician for this reason?.................... | 1 | 2 | 8 | 9 |
| C1.6. Do they affect you in your daily life?................................................. | 1 | 2 | 8 | 9 |
| C1.7. In relation to your memory, do you feel worse than other people of your age?............................................................................................ | 1 | 2 | 8 | 9 |

** C2. TELEPHONE INTERVIEW FOR COGNITIVE STATUS

| **Item** | | | | **Score** | | | | | | | |
| --- | --- | --- | --- | --- | --- | --- | --- | --- | --- | --- | --- |
|  |  | | |  | | | | | | | |
|  | Please tell me your first and last name: | | |  | | | | | | | |
| C2.1. | Name: | | | 0 | | | | 1 | | | |
| C2.2. | Last name: | | | 0 | | | | 1 | | | |
| C2.3. | What year is this? | | | 0 | | | | 1 | | | |
| C2.4. | What season of the year is this? | | | 0 | | | | 1 | | | |
| C2.5. | What month is it? | | | 0 | | | | 1 | | | |
| C2.6. | What day of the month is today? | | | 0 | | | | 1 | | | |
| C2.7. | What day of the week is today? | | | 0 | | | | 1 | | | |
|  | What is your mailing address? | | |  | | | | | | | |
| C2.8. | Country: | | | 0 | | | | 1 | | | |
| C2.9. | City: | | | 0 | | | | 1 | | | |
| C2.10. | Street: | | | 0 | | | | 1 | | | |
| C2.11. | Number: | | | 0 | | | | 1 | | | |
| C2.12. | Zip code: | | | 0 | | | | 1 | | | |
|  |  | | |  | | | | | | | |
|  |  | | |  | | | | | | | |
| C2.13. | Count backwards, one at a time, from 20 to 1: | | |  | | | | | | | |
|  | Attempt 1: | | |  | | | | | | | |
|  | If failed on attempt 1, Attempt 2: | | | 0 | | | 1 | | | 2 | |
|  | [If the participant counts correctly on attempt 1, a score of 2 is awarded. On the other hand, if he/she makes a mistake, he/she will be asked to try a second time; if he/she performs the test correctly on attempt 2, a score of 1 is awarded.] | | |  | | |  | | |  | |
|  |  | | |  | | | | | | | |
|  |  | | |  | | | | | | | |
|  | Next, I am going to read you a list of 10 words. Please listen carefully and try to retain them. When I am finished you will have to tell me as many words as you can remember in any order. Ready? The words are: CABIN, PIPE, ELEPHANT, CHEST, SILK, THEATER, CLOCK, WHIP, PILLOW, and GIANT. Now tell me which ones you remember: | | |  | | | | | | | |
| C2.14. | CABIN | | | 0 | | | | 1 | | | |
| C2.15. | PIPE | | | 0 | | | | 1 | | | |
| C2.16. | ELEFANT | | | 0 | | | | 1 | | | |
| C2.17. | CHEST | | | 0 | | | | 1 | | | |
| C2.18. | SILK | | | 0 | | | | 1 | | | |
| C2.19. | THEATER | | | 0 | | | | 1 | | | |
| C2.20. | CLOCK | | | 0 | | | | 1 | | | |
| C2.21. | WHIP | | | 0 | | | | 1 | | | |
| C2.22. | PILLOW | | | 0 | | | | 1 | | | |
| C2.23. | GIGANT | | | 0 | | | | 1 | | | |
|  | [One point is awarded for each word remembered. Plural forms are considered correct. Repetitions and intrusions are not penalized]. | | |  | | | |  | | | |
|  |  | | |  | | | | | | | |
|  |  | | |  | | | | | | | |
|  | Now I want you to subtract 7 from 7 starting at 100. That is, how much is 100 minus 7? Keep subtracting: | | |  | | | | | | | |
| C2.24. | 93 | | | 0 | | | | 1 | | | |
| C2.25. | 86 | | | 0 | | | | 1 | | | |
| C2.26. | 79 | | | 0 | | | | 1 | | | |
| C2.27. | 72 | | | 0 | | | | 1 | | | |
| C2.28. | 65 | | | 0 | | | | 1 | | | |
|  | [A correct answer is considered as long as the subtraction with respect to the previous number is equal to the subtraction of 7, regardless of whether the answer given was correct or not] | | |  | | | |  | | | |
|  |  | | |  | | | | | | | |
|  |  | | |  | | | | | | | |
| C2.29. | What tool is used to cut the paper? | | | 0 | | | | 1 | | | |
|  | [Only "scissors" or " cutter" answers are considered valid] | | |  | | | |  | | | |
|  |  | | |  | | | | | | | |
|  |  | | |  | | | | | | | |
| C2.30. | How many things are in a dozen? | | | 0 | | | | 1 | | | |
|  | [Only answer "12" is considered valid] | | |  | | | |  | | | |
|  |  | | |  | | | | | | | |
|  |  | | |  | | | | | | | |
| C2.31. | What is the name of the green spiky plant that lives in the desert? | | | 0 | | | | 1 | | | |
|  | [Only the answer "cactus" is considered valid] | | |  | | | |  | | | |
|  |  | | |  | | | | | | | |
|  |  | | |  | | | | | | | |
| C2.32. | What animal does the wool come from? | | | 0 | | | | 1 | | | |
|  | [Only the answers "sheep" or "lamb" are considered valid] | | |  | | | |  | | | |
|  |  | | |  | | | | | | | |
|  |  | | |  | | | | | | | |
| C2.33. | Repeat the following phrase: "NEITHER YES, NOR NO, NOR BUT" | | | 0 | | | | 1 | | | |
|  | [A point is awarded only if the repetition is correct on the first attempt] | | |  | | | |  | | | |
|  |  | | |  | | | | | | | |
|  |  | | |  | | | | | | | |
| C2.34. | Repeat the following phrase: "EPISCOPAL METHODIST" | | | 0 | | | | 1 | | | |
|  | [A point is awarded only if the repetition is correct on the first attempt] | | |  | | | |  | | | |
|  |  | | |  | | | | | | | |
|  |  | | |  | | | | | | | |
| C2.35. | Can you tell me the name of the Spanish Prime Minister? | | | 0 | | | | 1 | | | |
|  | [One point is awarded if the first and/or last name is correctly remembered] | | |  | | | |  | | | |
|  |  | | |  | | | | | | | |
|  |  | | |  | | | | | | | |
| C2.36. | And who is the King of Spain? | | | 0 | | | | 1 | | | |
|  | [One point is awarded only if it says "Felipe VI"] | | |  | | | |  | | | |
|  |  | | |  | | | | | | | |
|  |  | | |  | | | | | | | |
| C2.37. | Please tap your phone 5 times with one of your fingers: | | | 0 | | | | 1 | | | |
|  | [Two points are awarded if exactly 5 taps are done and one point if the individual taps a different number of times] | | |  | | | |  | | | |
|  |  | | |  | | | | | | | |
|  |  | | |  | | | | | | | |
| C2.38. | I´m going to tell you a word and want you to give me its opposite. For example, if I tell you hot, the opposite would be cold. What is the opposite of West? | | | 0 | | | | 1 | | | |
|  | [Only answers "East" or "Orient" are considered valid] | | |  | | | |  | | | |
|  |  | | |  | | | | | | | |
|  |  | | |  | | | | | | | |
| C2.39. | And what is the opposite of generous? | | | 0 | | | | 1 | | | |
|  | [Answers such as "stingy", "selfish" or similar are considered valid]. | | |  | | | |  | | | |
|  |  | | |  | | | | | | | |
|  |  | | |  | | | | | | | |
|  | Earlier I read you a list of 10 words, could you tell me as many as you can remember? | | |  | | | | | | | |
| C2.40. | CABIN | | | 0 | | | | 1 | | | |
| C2.41. | PIPE | | | 0 | | | | 1 | | | |
| C2.42. | ELEFANT | | | 0 | | | | 1 | | | |
| C2.43. | CHEST | | | 0 | | | | 1 | | | |
| C2.44. | SILK | | | 0 | | | | 1 | | | |
| C2.45. | THEATER | | | 0 | | | | 1 | | | |
| C2.46. | CLOCK | | | 0 | | | | 1 | | | |
| C2.47. | WHIP | | | 0 | | | | 1 | | | |
| C2.48. | PILLOW | | | 0 | | | | 1 | | | |
| C2.49. | GIGANT | | | 0 | | | | 1 | | | |
|  | [One point is awarded for each word remembered. Plural forms are considered correct. Repetitions and intrusions are not penalized]. | | |  | | | |  | | | |
|  |  | | |  | | | |  | | | |
|  |  | | |  | | | |  | | | |
| C2.50. | Spell the word WORLD backwards | 0 | 1 | | 2 | 3 | | | 4 | | 5 |
|  |  | | |  | | | |  | | | |

**D. PERCEIVED HEALTH AND QUALITY OF LIFE**

D1. SELF-PERCEIVED HEALTH

**In general, how would you rate your health status in the last 12 months?**

Very good..………………………………………………………………….. 1

Good……………………………………………………………………..…... 2

Neither good nor bad..……………………………………………..…. 3

Bad..……………………………………………………………………..…….. 4

Very bad..……………………………………………………………………. 5

Does not know…………………………………………………………….. 8

Does not answer …………………………………………………………. 9

D2. HEALTH THERMOMETER (*show image in Annex I*)

**On a scale from 0 to 100, where 0 represents the worst state of health you can imagine and 100 the best state of health you can imagine, please indicate how you think your state of health is today _________**

D3. PHYSICAL FITNESS ACTIVITY IN THE LAST TWO WEEKS

**During the last two weeks, what was the hardest physical activity you could do for at least two minutes?**

Very heavy (e.g., run at a fast pace)................................ 1

Heavy (e.g., jog, at a slow pace)..…................................. 2

Moderate (e.g., walk, at a fast pace).……........................ 3

Light (e.g., walk, at a medium pace)................................ 4

Very light (e.g., walk, at a slow pace)…..…….................... 5

None (e.g., unable to walk)………….……………………………… 6

D4. FEELINGS IN THE LAST TWO WEEKS

**During the past two weeks, how much have you been bothered by emotional problems such as feeling anxious, depressed, irritable or downhearted and sad?**

Not at all......…….............................................................. 1

Slightly............................................................................. 2

Moderately……….............................................................. 3

Quite a bit........................................................................ 4

Extremely.…….................................................................. 5

D5. DAILY ACTIVITIES IN THE LAST TWO WEEKS

**During the past two weeks, how much difficulty have you had in doing your usual activities or tasks, both inside and outside the house because of your physical and emotional health?**

None…………………............................................................. 1

A little bit………………........................................................ 2

Moderately difficult......................................................... 3

Highly difficult…............................................................... 4

Completely unable…………………....................................... 5

D6. SOCIAL ACTIVITIES IN THE LAST TWO WEEKS

**During the past two weeks, have your physical and emotional health limited your social activities with family, friends, neighbors or groups?**

Not at all…..………............................................................. 1

Slightly…..………………........................................................ 2

Moderately……….............................................................. 3

Quite a bit........................................................................ 4

Extremely....….................................................................. 5

D7. HEALTH STATUS CHANGE IN THE LAST TWO WEEKS

**How would you rate your health status now compared to two weeks ago?**

Much better..................................................................... 1

A little better…….............................................................. 2

About the same............................................................... 3

A little worse…….............................................................. 4

Much worse..................................................................... 5

D8. OVERALL HEALTH STATUS IN THE LAST TWO WEEKS

**During the past two weeks, how would you rate your health in general?**

Excellent.......................................................................... 1

Very good........................................................................ 2

Good................................................................................ 3

Fair……............................................................................. 4

Poor................................................................................. 5

D9. PAIN IN THE LAST TWO WEEKS

**During the last two weeks, how much pain have you had?**

None…………….................................................................. 1

Very light pain................................................................. 2

Light pain…...................................................................... 3

Moderate pain….............................................................. 4

Severe pain...................................................................... 5

D10. SOCIAL SUPPORT IN THE LAST TWO WEEKS

**During the past two weeks, was there anyone willing to help you if you needed help? For example: *felt nervous, lonely, or sad; *fell ill and had to stay in bed; *needed to talk to someone; *needed help with chores around the house; *needed help taking care of yourself.**

Yes, everyone was willing to help me………………............. 1

Yes, quite a few people................................................... 2

Yes, some people….......................................................... 3

Yes, someone……............................................................. 4

No one at all………............................................................ 5

D11. QUALITY OF LIFE IN THE LAST TWO WEEKS

**How have things been going for you in the last two weeks?**

Great, they couldn't be better………….............................. 1

Fairly well……................................................................... 2

Sometimes good, sometimes bad……………………………..... 3

Quite bad......................................................................... 4

Very bad, they could not have done any worse.............. 5

D12. LONELINESS

**How often have you felt lonely during the last year?**

Always or almost always................................................. 1

Often……….……................................................................. 2

Sometimes....................................................................... 3

Rarely to never……........................................................... 4

Does not know…………………………………………………………….. 8

Does not answer …………………………………………………………. 9

**E. FUNCTIONAL DEPENDENCE**

E1. DISABILITY

**Do you have a recognized disability?**

Yes………………………………………………………………………………. 1

No……………………………………………………………………………….. 2

Does not know…………………………………………………………….. 8

Does not answer …………………………………………………………. 9

E2. PERCENTAGE OF RECOGNIZED DISABILITY

**If you know, please tell us the percentage of your recognized disability**: _______ %

E3. DEPENDENCE

**Have you applied for an assessment of your dependency status?**

Yes, and I have the official resolution...……………………….. 1

Yes, but I am awaiting final resolution…………….…………... 2

No……………………………………………………………………………….. 3

Does not know…………………………………………………………….. 8

Does not answer …………………………………………………………. 9

E4. RECOGNIZED DEGREE OF DEPENDENCY

**If you know, please tell us the level of dependency you are recognized**: _______

* E5. VISUAL SENSORY IMPAIRMENTS

**Can you read the newspaper or watch TV normally (with glasses or contact lenses if you need them)?**

Yes, with no difficulty...................................................... 1

Yes, with some difficulty................................................. 2

E5.1. (*especify the reason* ___________________________)

Yes, with great difficulty.................................................. 3

E5.2. (*especify the reason* ___________________________)

No, unable to do it…........................................................ 4

* E6. SENSORY HEARING IMPAIRMENTS

**Can you hear normally what is said in a conversation of three or four people (with a hearing aid if needed)?**

Yes, with no difficulty...................................................... 1

Yes, with some difficulty................................................. 2

E6.1. (*especify the reason* ___________________________)

Yes, with great difficulty.................................................. 3

E6.2. (*especify the reason* ___________________________)

No, unable to do it…........................................................ 4

* E7. KATZ INDEX

**Now I am going to ask you some questions about ordinary activities in anyone's life. Tell me if you are able to...**

| E7.1. BATHING |  |
| --- | --- |
| Independence. Bath self completely or need help in bathing only a single part of the body such as the back, genital area or disabled extremity…………..……….. | 1 |
| Dependence. Need help with bathing more than one part of the body, getting in or out of the tub or shower. Require total bathing………………………………….…… | 0 |
| E7.2. DRESSING |  |
| Independence. Get clothes from closets and drawers and put on clothes and outer garments complete with fasteners. May have help tying shoes…………….. | 1 |
| Dependence: Need help with dressing self or need to be completely dressed………………………………………………………………......……………………………………… | 0 |
| E7.3. TOILETING |  |
| Independence. Go to the toilet, get on and off, arrange clothes, clean genital area without help………………………………………………………………………………..…………. | 1 |
| Dependence. Need help transferring to the toilet, cleaning self or uses bedpan or commode………….………………………………………………..……………………………………… | 0 |
| E7.4. TRANSFERRING |  |
| Independence. Move in and out of bed or chair unassisted. Mechanical transfer aids are acceptable……………………………………………………………………………………….... | 1 |
| Dependence. Need help in moving from bed to chair or requires a complete transfer…………………………………………………………………………………………………………… | 0 |
| E7.5. CONTINENCE |  |
| Independence. Exercise complete self control over urination and defecation………………………………………………………………………..…………………………….. | 1 |
| Dependence. Are partially or totally incontinent of bowel or bladder……..……… | 0 |
| E7.6. FEEDING |  |
| Independence. Get food from plate into mouth without help. Preparation of food may be done by another person……………………………………………………………… | 1 |
| Dependence. Need partial or total help with feeding or require parenteral feeding………………………………………………….………………………………………………………… | 0 |

**F. LIFESTYLES AND HABITS**

F1. WEIGHT

**Approximately how much do you weigh without shoes and clothes?** _______ kg

F2. HEIGHT

**Approximately how tall are you without shoes?** _______ cm

F3. HOURS OF SLEEP PER DAY

**Could you tell me approximately how many hours you sleep per day including naps?** _______ hours per day

F4. SLEEP QUALITY

**And how do you sleep? Would you say you sleep very well, well, fairly, poorly or very poorly?**

Very well.…………………………………………………………………….. 1

Well………………………………………………………………………..…... 2

Fair…………………………………………………………………………..…. 3

Bad..……………………………………………………………………..…….. 4

Very bad………………………………………………………………………. 5

Does not know…………………………………………………………….. 8

Does not answer …………………………………………………………. 9

F5. SMOKING

**Can you tell me if you currently smoke?**

Yes, daily……….................................................................. 1

F5.1. (*specify, how many years have you been smoking?* _________)

Yes, but not daily………….………………….…………………………. 2

F5.2. (*specify, how many years have you been smoking?* _________)

Not currently, but previously……………… ……………………… 3

F5.3. (*specify, for how many years did you smoke?* _________)

F5.4. (*specify, how many years ago did you quit? _________*)

Do not smoke, and never smoked on a regular basis ….. 4

Does not know…………………………………………………………….. 8

Does not answer …………………………………………………………. 9

F6. ALCOHOL CONSUMPTION

**Can you tell me if you currently drink any alcohol?**

Yes, at least five times a week………….............................. 1

F6.1. (*specify, what type of alcoholic beverage?* _________)

F6.2. (*specify, how much alcoholic beverage?* _________)

F6.3. (*specify, for how many years?* _________)

Not currently, but previously……………. ………………..……… 2

F6.4. (*specify, for how many years did you drink?* _________)

F6.5. (*specify, how many years ago did you quit? _________*)

Does not drink, and never drunk on a regular basis ..….. 3

Does not know…………………………………………………………….. 8

Does not answer …………………………………………………………. 9

F7. FEEDING

**How often do you consume the following food groups per week?**

|  | **0 days** | **1-2 days** | **3 days** | **4-6 days** | **Daily** |
| --- | --- | --- | --- | --- | --- |
| F7.1. Vegetables, salads and greens……………………….…....... | 0 | 1 | 2 | 3 | 4 |
| F7.2. Fresh fruit (excluding natural juices)………..…………….. | 0 | 1 | 2 | 3 | 4 |
| F7.3. Extra virgin olive oil ……………………………………………..…… | 0 | 1 | 2 | 3 | 4 |
| F7.4. Legumes…..…………………………………………………………….. | 0 | 1 | 2 | 3 | 4 |
| F7.5. Fish…………..……………………………………………………………. | 0 | 1 | 2 | 3 | 4 |
| F7.6. Nuts………………………………………………………………………… | 0 | 1 | 2 | 3 | 4 |
| F7.7. Coffee (with or without caffeine)……………………………… | 0 | 1 | 2 | 3 | 4 |

F8. DAILY ACTIVITIES

**How often do you engage in the following leisure activities per week?**

|  | **0 days** | **1-2 days** | **3 days** | **4-6 days** | **Daily** |
| --- | --- | --- | --- | --- | --- |
| F8.1. Walking for more than 10 minutes at a time ………….… | 0 | 1 | 2 | 3 | 4 |
| F8.2. Other physical activity (gymnastics, tai chi, etc.)…..…  (*specify* __________________________) | 0 | 1 | 2 | 3 | 4 |
| F8.3. Creative activities (painting, writing, etc.)………………. | 0 | 1 | 2 | 3 | 4 |
| F8.4. Going out with friends for a walk, having a coffee….. | 0 | 1 | 2 | 3 | 4 |
| F8.5. Doing pastimes (crossword puzzles, sudokus, etc.)….. | 0 | 1 | 2 | 3 | 4 |
| F8.6. Attending courses or workshops at senior centers……. | 0 | 1 | 2 | 3 | 4 |
| F8.7. Going to the cinema, theater, concerts, etc………….… | 0 | 1 | 2 | 3 | 4 |
| F8.8. Listening to music…………………………………………………… | 0 | 1 | 2 | 3 | 4 |
| F8.9. Watching TV; listen to the radio…………………………….… | 0 | 1 | 2 | 3 | 4 |
| F8.10. Reading (newspaper, books or magazines)……………. | 0 | 1 | 2 | 3 | 4 |
| F8.11. Use of technology (cell phone, computer)….………… | 0 | 1 | 2 | 3 | 4 |

**ANNEX I**

The best state of health you can imagine

The worst state of health you can imagine

**HEALTH THERMOMETER**

To help people describe how good or bad their state of health is, we have drawn a scale similar to a thermometer on which the best state of health you can imagine is marked with a 100 and the worst state of health you can imagine is marked with a 0.

We would like you to indicate on this scale, in your opinion, how good or bad your state of health is TODAY. Please draw a line from the box where it says "**Your state of health TODAY**" to the point on the thermometer that in your opinion indicates how good or bad your state of health is TODAY.

**Your state of health TODAY**
